# Orchid diversity: Spatial and climatic patterns from herbarium records

**DOI:** 10.1002/ece3.4598

**Published:** 2018-10-30

**Authors:** Anne C. Gaskett, Rachael V. Gallagher

**Affiliations:** ^1^ School of Biological Sciences The University of Auckland Auckland New Zealand; ^2^ Department of Biological Sciences Macquarie University Sydney New South Wales Australia

**Keywords:** Australian Virtual Herbarium, biogeography, collecting effort, natural history collections, niche breadth, Orchidaceae, protected areas, species richness

## Abstract

**Aim:**

We test for spatial and climatic patterns of diversification in the Orchidaceae, an angiosperm family characterized by high levels of species diversity and rarity. Globally, does orchid diversity correlate with land area? In Australia, does diversity correlate with herbarium collecting effort, range size, or climate niche breadth? Where are Australia's orchids distributed spatially, in protected areas, and in climate space?

**Location:**

Global, then Australia.

**Methods:**

We compared orchid diversity with land area for continents and recognized orchid diversity hotspots. Then, we used cleaned herbarium records to compare collecting effort (for Australian Orchidaceae vs. all other plant families, and also among orchid genera). Spatial and climate distributions were mapped to determine orchids’ coverage in the protected area network, range sizes, and niche breadths.

**Results:**

Globally, orchid diversity does not correlate with land area (depauperate regions are the subantarctic: 10 species, and northern North America: 394 species). Australian herbarium records and collecting effort generally reflect orchid species diversity (1,583 spp.), range sizes, and niche breadths. Orchids are restricted to 13% of Australia's landmass with 211 species absent from any protected areas. Species richness is the greatest in three biomes with high general biodiversity: Temperate (especially southwest and southeast Australia), Tropical, and Subtropical (coastal northern Queensland). Absence from the Desert is consistent with our realized climate niche—orchids avoid high temperature/low rainfall environments. Orchids have narrower range sizes than nonorchid species. Highly diverse orchid genera have narrower rainfall breadths than less diverse genera.

**Main conclusions:**

Herbarium data are adequate for testing hypotheses about Australian orchids. Distribution is likely driven by environmental factors. In contrast, diversification did not correlate with increases in range size, rainfall, or temperature breadths, suggesting speciation does not occur via invasion and local adaptation to new habitats. Instead, diversification may rely on access to extensive obligate symbioses with mycorrhizae and/or pollinators.

## 1 INTRODUCTION

Orchidaceae is the most diverse flowering plant family with approximately 25,000 species worldwide, comprising ~10% of all angiosperms (Cribb, Kell, Dixon, & Barrett, 2003). Orchids have colonized all vegetated continents and several subantarctic islands, have one of the broadest latitudinal ranges of all plant families, and occupy a wide range of habitats in epiphytic, terrestrial, and even subterranean forms (Cribb et al., 2003; Givnish et al., 2016; Skotnicki et al., 2009). Despite this diversity, many orchids are naturally rare, exacerbated by human activities especially habitat destruction and degradation (including via climate change), and illegal collecting (Cribb et al., 2003; Reiter et al., 2016; Wraith & Pickering, 2017). Some of these threatening processes could potentially be mitigated if orchid species are present in protected areas or can be translocated (Swarts & Dixon, 2009b; Reiter et al., 2016, but see Wraith & Pickering, 2017).

Orchid distributions and rarity are also likely to be affected by their many intimate, and often obligate, multispecies relationships (Pemberton, 2010). For example, orchids rely on mycorrhizal fungi for nutrients, especially as seedlings (Rasmussen & Rasmussen, 2009). The degree of specialization in these orchid–mycorrhizae relationships varies, with implications for orchid distributions and rarity (Davis, Phillips, Wright, Linde, & Dixon, 2015; Jacquemyn, Brys, Waud, Busschaert, & Lievens, 2015). In turn, the distributions of mycorrhizal fungi depend largely upon edaphic conditions such as soil moisture, pH, nutrients, and organic content (McCormick & Jacquemyn, 2014; Nurfadilah, Swarts, Dixon, Lambers, & Merritt, 2013). Orchid–pollinator relationships are often unusual and sometimes highly specialized (Gaskett, 2011; Xu, Schlüter, & Schiestl, 2012), and the distribution and availability of these specialized pollinators are critical for successful orchid conservation efforts (Reiter et al., 2017, 2016). The insects that pollinate orchids have additional symbioses with other taxa, for example, food plants and larval hosts (Brown & Phillips, 2014; Kelly, Toft, & Gaskett, 2013; Reiter, Lawrie, & Linde, 2018). These extensive, interconnected relationships could limit the distribution of orchids to sites with high general biodiversity (Pemberton, 2010). If orchids are potential bioindicators of general diversity (Newman, Ladd, Batty, & Dixon, 2007), mapping their presence/absence in protected areas gives insight into the distribution and conservation of not just orchids, but a broader network of symbiotic insect, plant, and fungal partners.

Despite strong research interest in orchid biology and conservation, there are few comparative investigations of the distribution, diversity, and niche characteristics of orchids at continental scales. A single, landscape scale study is available, addressing orchid diversity, habitat, and climate in China (Zhang et al., 2015). Most orchid species (90%) were distributed across only 2.7% of China's landmass, coinciding with regions of high general plant diversity. Nearly, all species were covered by nature reserves (~97% of 1,449 species), and patterns of orchid species richness were best explained by abiotic factors including net primary productivity (24.5%) and moisture index (16.2%).

Some studies address orchid ecological preferences at regional scales, often with a conservation perspective (Phillips, Hopper, & Dixon, 2010; Tsiftsis, Tsiripidis, Karagiannakidou, & Alifragis, 2008). For example, for orchids in the southwest Australian Floristic Region (SWAFR), presence–absence data from herbarium records were tested against biogeographic provinces, ecological factors including pollination strategy, and climate variables, to explore patterns in species rarity (Phillips, Brown, Dixon, & Hopper, 2007, 2011 ). Taxon‐specific distribution analyses have been performed for some Australian orchid species by coupling field or historical orchid abundances with climate and environmental data, for example, for rare species such as *Cryptostylis hunteriana* (Clark, deLacey, & Chamberlain, 2004) and underground *Rhizanthella gardneri* (Bougoure, Brundrett, Brown, & Grierson, 2008), and the diverse genus *Pterostylis* (Janes, Steane, & Vaillancourt, 2010). However, despite the wide accessibility of data on plant distribution, phenology, and climate (Hijmans & van Etten, 2012; Lavoie, 2013; Willis et al., 2017), studies of orchids at continental and global scales are surprisingly lacking. Baseline ecological information about where orchids occur and why is needed for understanding evolution and diversity, and more pragmatic goals such as conservation and climate change adaptation planning.

In this study, we analyze the patterns of occurrence of 1,538 native Australian orchid taxa using a dataset of 174,592 digitized herbarium records and long‐term climate averages. Herbarium records are effective for exploring changes in orchid distributions (Kull & Hutchings, 2006) and pollination rates (Pauw & Hawkins, 2011; Robbirt, Davy, Hutchings, & Roberts, 2011), and evidence of phenological cues that track climate and the consequences for this under future climate change (Gallagher, Hughes, & Leishman, 2009). Orchids originated in Australia 112 Mya before dispersing globally (Givnish et al., 2015), making it the ideal place to investigate long‐standing patterns of diversity. The current Australian orchid flora is almost entirely within the tribe Diurideae, which arose in Africa from Neotropical origins ~50 Mya, then reinvaded Australia (Givnish et al., 2015, 2016; Kores et al., 2001; Weston, Perkins, Indsto, & Clements, 2014). This shared origin means that comparative analyses are unlikely to be compromized by phylogenetic elements. Although Australia is occasionally considered depauperate in orchid species given its land area (Dafni & Bernhardt, 1990; van der Cingel, 2001), other sources indicate that orchid diversity and endemism are high (~1,200–1,700 species; Hopper, 2009; Swarts & Dixon, 2009b; WCSP, 2016), and may rank alongside well‐recognized orchid hotspots such as South and Central America and Southeast Asia (Cribb et al., 2003). Australian environments are highly varied (montane meadows, arid grasslands, tropical rainforests), and digitized vouchered herbarium collections are plentiful and freely available online (CHAH, 2009).

Here, we use herbarium data to explore orchid diversity relative to land area, collecting effort, spatial and climatic distributions, and the protected area network. We test the following hypotheses: (a) Globally, Australia is relatively depauperate in orchid diversity per unit land area, (b) herbarium collecting effort for orchids is similar to other highly diverse Australian plant families, (c) orchid species are adequately represented in protected areas, (d) orchid richness differs between biomes within Australia, (e) orchids from highly diverse genera have larger range sizes and occur across a wider breadth of temperature and rainfall conditions than those from less diverse genera.

## 2 METHODS

### 2.1 Land area and orchid diversity

Numbers of orchid species per continent and for known orchid diversity hotspots were obtained from the World Checklist of Selected Plant Families (WCSP, 2016), the World Geographical Scheme for Recording Plant Distributions (Brummitt, 2001; Hopper & Gioia, 2004; Zhang et al., 2015). Land areas were from the 2015 United Nations Demographic Yearbook (UN Statistics Division, 2015) and the Island Directory (Dahl, 1991). We performed linear regressions for the number of orchid species (log_10_ transformed) and area in km^2^ for (a) continents and (b) known orchid diversity hotspots. To test whether Australia is depauperate, we compared the number of orchid species per km^2^ between Australia and the other continents via ANOVA. Continental comparisons were performed both with and without Antarctica since Antarctic orchids are restricted to small, ice‐free subantarctic islands comprising just 0.35% of Antarctica's area (Dahl, 1991).

### 2.2 Species occurrence records

We used records from Australia's Virtual Herbarium (AVH; via the Atlas of Living Australia [ALA] Data Download Portal; https://collections.ala.org.au/) to characterize the distributional range of native Australian orchid species. The AVH is the largest source of Australian floral distribution data and is based on digitized records of vouchered specimens from Australia's nine major herbaria (CHAH, 2009). This preliminary dataset was cleaned by removing records which were (a) not identified to species level (i.e., consisting only of a genus name and the epithet “sp.”); (b) collected in a country other than Australia; (c) lacking in georeferencing information (latitude and longitude coordinates); (d) duplicates (i.e., nonunique combinations of latitude, longitude, and species name); (e) cultivated; (f) hybrid combinations between species; and (g) not native (i.e., introduced species identified in both (Randall, 2007) and using the tag “naturalized” provided in the Australian Plant Census [APC] https://biodiversity.org.au/nsl/services/apc). The dataset thus consisted of 174, 592 occurrence records for 1,538 orchid species (Supporting Information Table S1.1 in Appendix S1). Records in the ALA provide the name submitted at the time of collection, plus a corrected name where relevant. Here, we use the corrected name, reflecting the accepted names in current literature.

Use of herbarium records for orchid studies is hindered by taxonomic uncertainty, often due to orchids’ capacity to readily hybridize and speciate. Many Australian orchid groups have undergone revisions, sometimes involving controversial splitting of genera (Hopper, 2009; Jones, 2006) and strict taxonomic rules may underrepresent true orchid species richness. For example, in 2014, the Australian Virtual Herbarium listed 1,538 Australian orchid species and 174,591 collection records, whereas in 2017, this dropped to only 212 species and 25,334 records. This is largely because the Australian Virtual Herbarium is now taxonomically aligned to the APC and its very conservative nomenclatural rules. In 2017, the APC listed 795 orchid species, but only 333 were APC concept names (confirmed taxonomic entities; CANBR, 2017). The APC also draws from the Flora of Australia, for which the Orchidaceae are yet to be treated (ABRS, 2017). The 2017 AVH data are inconsistent with orchid species richness data from the Kew Botanic Gardens World Checklist of Selected Plant Families (1,529 spp. in 2014, 1,628 spp. in 2017; WCSP, 2016), and the checklist used and developed by Australian orchid researchers and taxonomists (1,872 spp. including tagnames; Backhouse, Bates, Brown, & Copeland, 2016). Therefore, in our analyses, we use the 2014 Australian Virtual Herbarium data and aim to explore general patterns in orchid distributions, diversity, and herbarium collection.

### 2.3 Do herbarium records and collecting effort reflect species diversity?

We tested whether herbarium records represent Australian plant diversity with linear regressions comparing the number of species versus the number of collecting records for (a) all plant families or (b) all orchid genera (data were log_10_ transformed to approximate normality). Collecting effort (log_10_ [number of records/number of species]) was compared between the Orchidaceae and the other top 10 most diverse plant families using ANOVA.

### 2.4 Distribution: Spatial and climatic

We mapped patterns of orchid richness by projecting all occurrence records into a 0.5° × 0.5° equal area grid of the Australian continent and counting the number of orchid taxa (either species or genera) in each grid cell. All mapping was performed in R version 3.0.2 (R Foundation for Statistical Computing, Vienna, Austria) using the “*raster*” package (Hijmans & van Etten, 2012) and in ArcGIS v. 10.2 (Environmental Systems Research Institute, Redlands, CA, USA). We determined range size (the area of occupancy in km^2^) by counting the number of 10 km × 10 km (100 km^2^) equal area grid cells occupied by the species across Australia. We compared the log_10_ transformed range sizes of orchid species and other higher plants (angiosperm and gymnosperm species) using ANOVA with post hoc Tukey tests.

We overlaid occurrence records with the 2014 Collaborative Australian Protected Area Database (https://www.environment.gov.au/land/nrs/science/capad) and extracted the number of species in each protected area. We used this to then count how many species were (a) absent from all protected areas, (b) present only in protected areas, or (c) found in both protected and unprotected areas.

**Figure 1:**
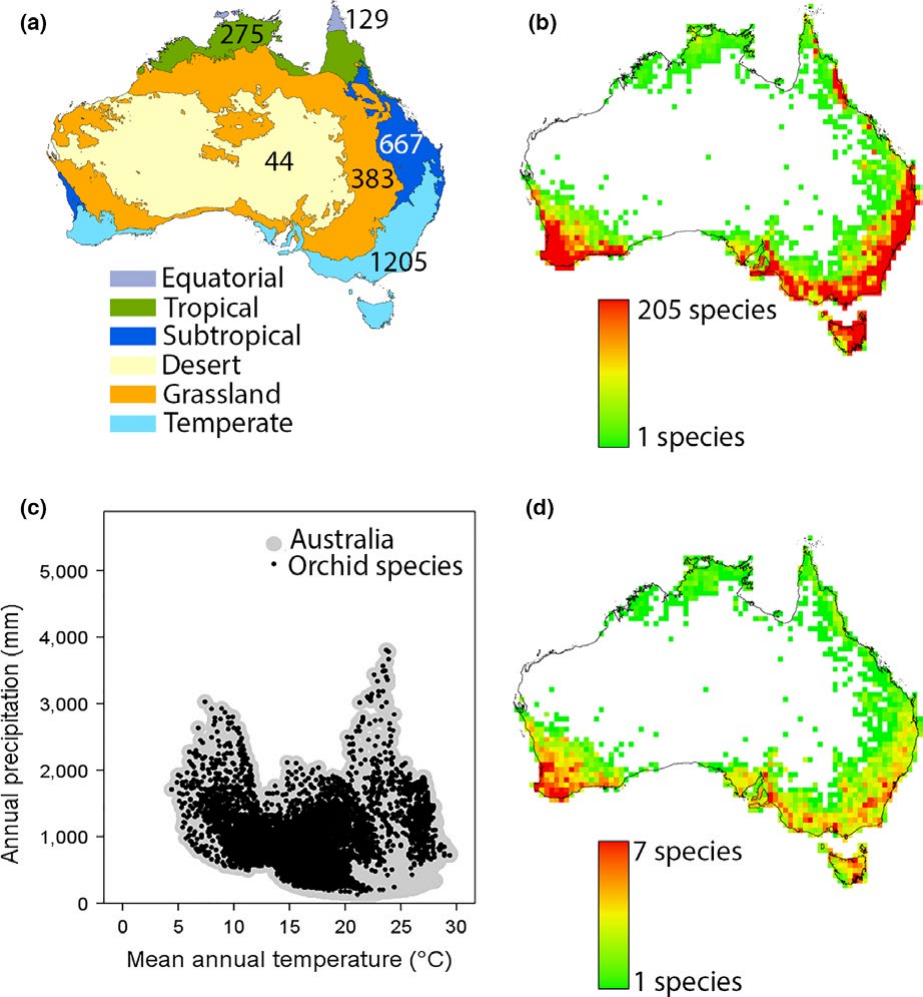
Orchid species richness across Australia. (a) The biomes of Australia (Koppen Climate Classification; Bureau of Meteorology, 2006). Values indicate the number of orchid species found in each biome. (b) The number of orchid species recorded in each 0.5° grid cell across Australia based on records from Australia's Virtual Herbarium (http://http::http:////avh.chah.org.au/) for 1,538 orchid species. (c) The realized climate niche of orchid species in Australia. Black circles represent location records in a climate space defined by mean annual temperature and annual precipitation. Large gray circles represent all the available climate space for these two variables across Australia. Climate data were extracted at a 5 arc minute resolution using baseline data for the period 1950–2000 available from the Worldclim dataset (http://http::http:////www.worldclim.org/; Hijmans et al., 2005). (d) The number of orchid genera relative to the number of orchid species in each 0.5° grid cell

We calculated the richness of orchid genera and species in different biomes across Australia by overlaying a shapefile of the Köppen climate classification (Bureau of Meteorology, 2006) on all orchid species occurrences and extracting a count of the number of species present in each biome. The Australian Köppen climate classification (Bureau of Meteorology, 2006; Stern, Hoedt, & Ernst, 2000) divides Australia into six biomes (desert, equatorial, grassland, subtropical, temperate, tropical; Figure 1a).

We visualized the climate conditions that orchids occupy across Australia by extracting values of mean annual temperature (°C; MAT) and annual rainfall (mm; AP) for each orchid occurrence from gridded climate datasets and overlaying them onto a two‐dimensional bi‐plot of the total available climate space for Australia. Total Australian climate space was determined by extracting MAT and AP for all grid cells across the continent at a 5‐arc minute resolution. Climate values were extracted from interpolated long‐term average conditions (1950–2000) represented by the Worldclim dataset (Hijmans, Cameron, Parra, Jones, & Jarvis, 2005).

### 2.5 Orchid diversification: Spatial and climatic patterns

To explore climatic factors associated with orchid diversity, we characterized the range of temperature and rainfall conditions occupied by each orchid species across its range (i.e., niche breadth). Niche breadths were determined by extracting data on MAT and AP for each occurrence record using the Worldclim dataset and calculating the range of conditions occupied by each species. Then, we compared the log_10_ transformed niche breadths between the five most highly diverse orchid genera, and all the remaining orchid genera, using ANOVA with post‐hoc Tukey tests.

## 3 RESULTS

### 3.1 Land area and orchid diversity

**Figure 2:**
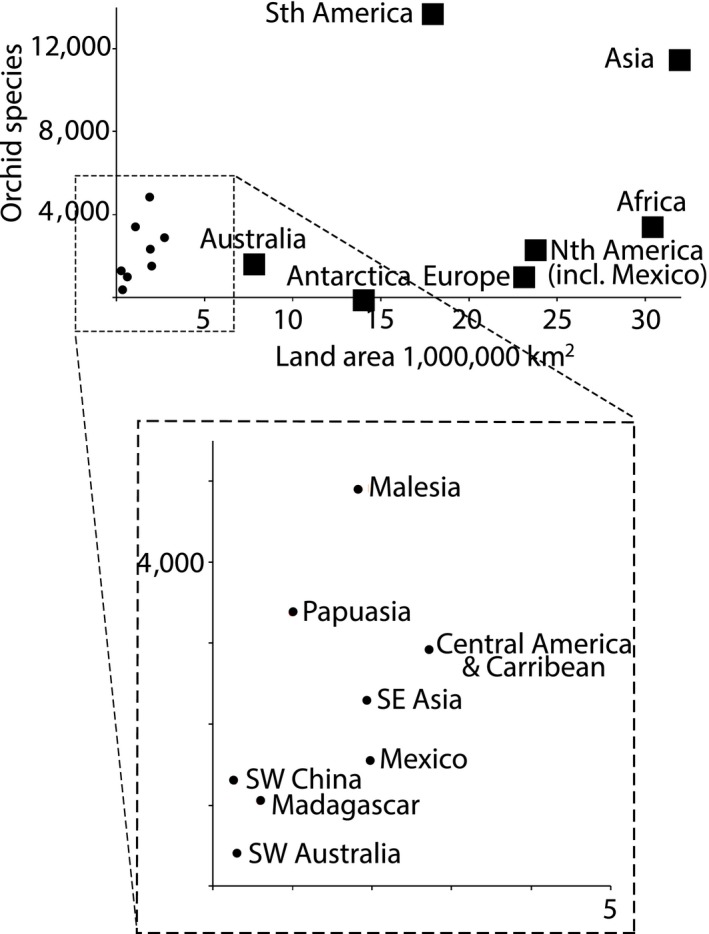
Orchid species richness versus land area for continents (squares) and recognized orchid diversity hotspots (dots). Orchid diversity data are from the Kew Botanic Gardens World Checklist of Selected Plant Families (WCSP, 2016). Land areas are from the 2015 United Nations Demographic Yearbook (UN Statistics Division, 2015). Floristic biogeographic zones are according to The World Geographic Scheme for Recording Plant Distributions, Edition 2 (Brummitt, 2001). Malesia includes Borneo, Cocos (Keeling) Islands, Jawa, Lesser Sunda Islands, Malaya, Maluku/Moluccas, The Philippines, Sulawesi, Sumatera/Samatra, and Christmas Island. Papuasia includes the Bismarck Archipelago, New Guinea, and the Solomon Islands.

There was no significant relationship between orchid diversity and land area for continents (Figure 2; with Antarctica: *R* ^2^ = 0.199, *F*_1,7_ = 1.493, *p* = 0.268; without Antarctica: *R* ^2^ = 0.080, *F*_1,6_ = 0.434, *p* = 0.539) or recognized orchid hotspots (*R* ^2^ = 0.431, *F*_1,7_ = 4.538, *p* = 0.077). Australian orchid density (number of species per km^2^) is not significantly different to that of the other continents (ANOVA, with Antarctica: *F*_1,7_ = 0.001, *p* = 0.972; without Antarctica: *F*_1,6_ = 0.005, *p* = 0.945).

### 3.2 Do herbarium records and collecting effort reflect species diversity?

**Figure 3:**
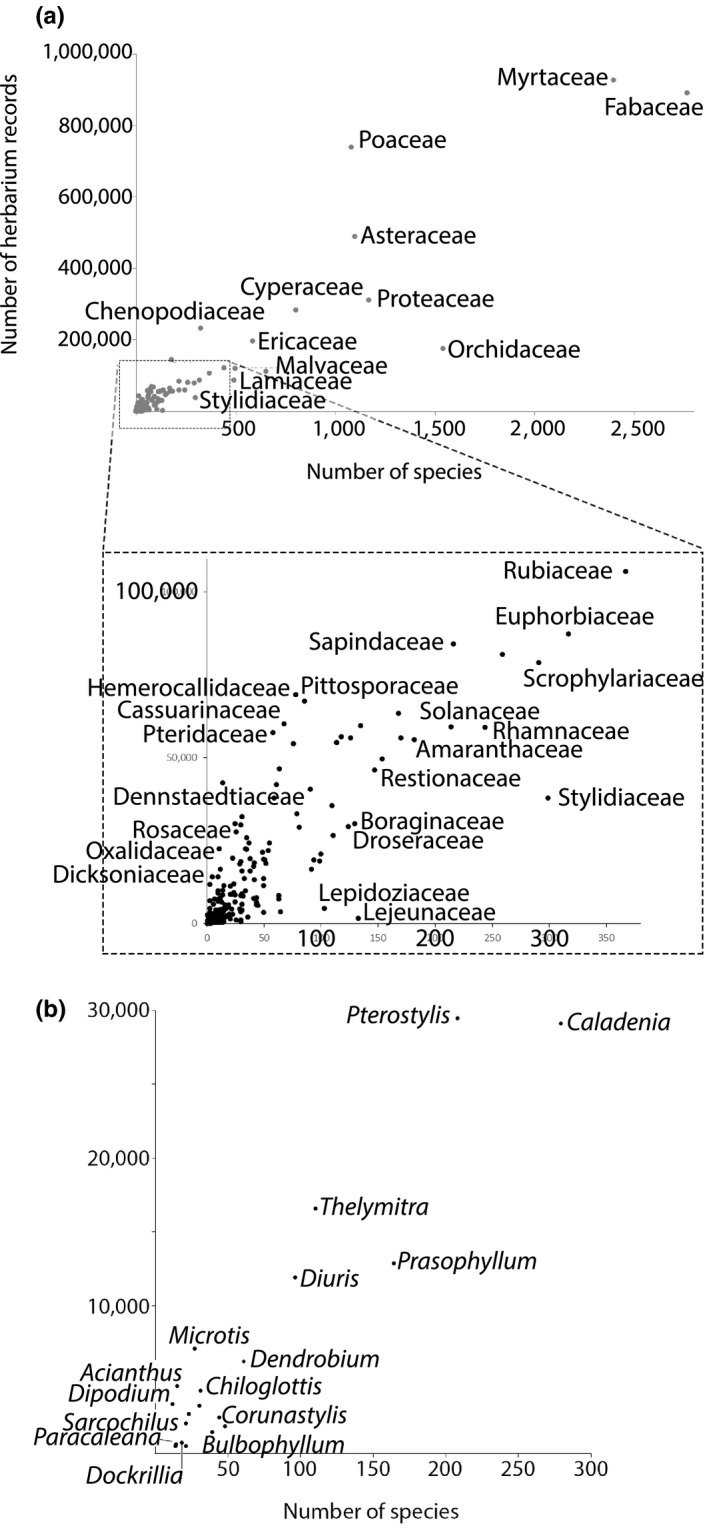
(a) Collecting effort for 301 Australian plant families, based on records from Australia's Virtual Herbarium (http://http::http:////avh.chah.org.au/). (b) Collecting effort for Australia's 20 most speciose orchid genera, based on records from Australia's Virtual Herbarium (http://http::http:////avh.chah.org.au/)

Herbarium records indicate that orchid species comprise 6.77% of the Australian flora as captured in cleaned digitized specimens from the Australian Virtual Herbarium (1,540 of 22,731 spp.). The Orchidaceae are the third most speciose family, and the third most collected (Supporting Information Table S2.1 in Appendix S2; Figure 3a). For all families, there was a significant linear relationship between species diversity and the number of herbarium records (*R* ^2^ = 0.751, *F*_1,300_ = 900.875, *p* < 0.0001; Figure 3a). However, collecting effort (number of herbarium records per species) was marginally, but significantly, lower for the Orchidaceae than for the other top 10 most diverse plant families (ANOVA: *F*_1,10_ = 5.243, *p* = 0.048; Figure 3a).

Collections have been made for 120 Australian orchid genera, and the most highly collected genera are also the most speciose (terrestrial genera *Caladenia* and *Pterostylis*; Figure 3b). There was a significant positive linear relationship between the diversity of an orchid genus and number of herbarium records (*R* ^2^ = 0.589, *F*_1,119_ = 168.978, *p* < 0.001; Supporting Information Table S2.2 in Appendix S2).

### 3.3 Distribution: Spatial and climatic

Australia's orchid species are restricted to 13% of the landmass. Of the 10,812 protected areas in Australia, 2,809 have at least one orchid species present. Of the 1,538 orchid species in our study, 211 (13.7%) are found only outside of the protected area network and 124 (8.1%) are found only within the protected area network (see Supporting Information Table S1.1 in Appendix S1).

**Figure 4:**
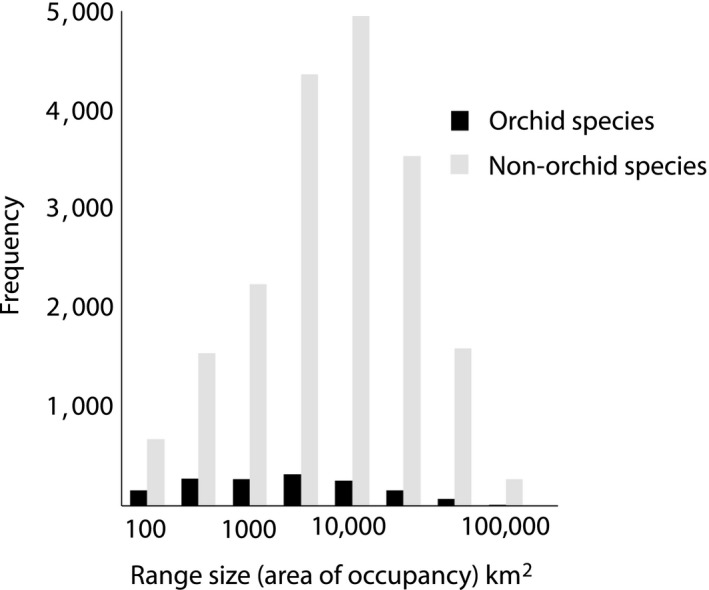
Frequency distributions of range size (km^2^) for orchid and nonorchid species (angiosperms and gymnosperms)

Australia's orchid diversity hotspots correspond with three biomes (Figure 1a,b): Temperate (in particular, southwest Western Australia and southeast Australia), Tropical, and Subtropical (combined areas of coastal northern Queensland). Orchids are almost absent from the Desert biome in the center of Australia and scarce in the Grassland biome. Correspondingly, the realized climate niche indicates that orchids are found in almost all of Australia's climate space, except where high temperatures correlate with low rainfall (i.e., the desert biome; Figure 1c). When we compared range sizes (occupancy in km^2^), the orchids occupied significantly smaller ranges than for other angiosperms, or gymnosperms (Figure 4; mean ± *SD* for orchids: 5,256.54 ± 11,471.00 km^2^; other plants: 8,501.83 ± 14,853.17; *F*_1,20,723_ = 423.814, *p* < 0.001).

### 3.4 Orchid diversification: Spatial and climatic patterns

Orchid diversification (as measured by the number of species per genus in each grid cell) is the greatest in the regions of highest general orchid richness, which occur in the southeast and southwest corners of the continent and the wet tropics bioregion in northern Queensland (Figure 1b,d). The number of herbarium collection records correlates positively with orchid species range sizes (*F*_358,1536_ = 45.467, *p* < 0.001), temperature breadth (*F*_358,1536_ = 6.998, *p* < 0.001), and rainfall breadths (*F*_358,1536_ = 6.564, *p* < 0.001).

The top five most speciose orchid genera had different range sizes to the other orchid genera (*F*_5,1536_ = 5.493, *p* < 0.001), but Tukey post hoc tests reveal this was driven largely by the only genus with an exclusively nectar rewarding pollination strategy, *Prasophyllum*, which had a smaller range than the other orchids (*p* values >0.05; Table 1). The top five most speciose genera all had significantly narrower rainfall breadths than the rest of the orchid genera (Table 1; rainfall *F*_5,1536_ = 28.689, *p* < 0.001, post hoc Tukey tests all *p* values <0.05). Two of the genera also had significantly narrower temperature breadths than other orchid genera (*F*_5,1536_ = 8.863, *p* < 0.001; *Caladenia* and *Prasophyllum* Post hoc Tukey test *p* values <0.001; but *Diuris, Thelymitra,* and* Pterostylis* Post hoc Tukey test *p* values >0.05).

**Table 1:** Temperature and precipitation breadths for the top five most speciose orchid genera in Australia and all other orchid genera

Orchid genera	*n* species	Range size (km^2^)	Temperature breadth (°C)	Rainfall breadth (mm)
Top five most speciose genera				
*Caladenia*	279	48,512.5 ± 6,535.0	3.33 ± 0.20	500.15 ± 27.37
*Pterostylis*	208	63,461.5 ± 9,944.91	4.52 ± 0.29	656.18 ± 43.26
*Prasophyllum*	164	31,036.6 ± 4,996.89	3.53 ± 0.27	545.19 ± 43.16
*Thelymitra*	110	70,818.2 ± 11,644.99	4.95 ± 0.38	640.59 ± 61.08
*Diuris*	96	55,718.8 ± 10,975.30	4.46 ± 0.39	620.10 ± 48.30
Remaining orchid genera (*n* = 115)	709	52,689.70 ± 4,440.88	4.92 ± 0.149	965.55 ± 29.199

Note

Values are mean ± *SE*.

## 4 DISCUSSION

Australia is sometimes described as having low orchid diversity given the land area, perhaps due to the large expanses of desert (Dafni & Bernhardt, 1990; van der Cingel, 2001). Our global comparison found no evidence for this, despite orchids only occupying 13% of the Australian landmass (cf. 2.7% of China; Zhang et al., 2015). Instead, we found North America surprisingly depauperate given its large land size, especially when Mexican orchid species are considered separately (Mexico = 1,560 spp., USA, Canada and Alaska = 394 spp.; Krupnick, McCormick, Mirenda, & Whigham, 2013; WCSP, 2016).

Despite their diversity, widespread appeal, and conservation status (Brundrett, 2007; Swarts & Dixon, 2009a), orchids are less collected than the other similarly diverse plant families in Australian herbarium records. Species rarity can hinder collection, but in general, museum collections tend to overrepresent rare species (Garcillán & Ezcurra, 2011; Guralnick & Van Cleve, 2005). As yet, there are no other studies of whether herbarium collections and collecting effort represent the natural diversity and abundance of orchids. It may be that botanists are reluctant to collect orchids because they are aware of orchids’ low rates of pollination, fruit set, and recruitment (Brundrett, 2007). Researchers and enthusiasts may also not wish to publicize orchid species and sites as this can lead to overexploitation by enthusiasts or illegal harvesting (Wraith & Pickering, 2017). Possibly, CITES regulations intended to prevent international trade in orchids may also deter some international scholarship and collecting efforts (Roberts & Solow, 2008).

Herbarium records often accurately reflected relative species diversity within orchid genera (e.g., *Caladenia* and *Pterostylis*), although some genera were surprisingly well‐collected given their lesser diversity, for example, *Acianthus* (15 spp., 4,584 records) and *Microtis* (27 spp., 720 records). These genera have some of the broadest areas of occupancy (mean values: *Acianthus* 12,420 km^2^, *Microtis* = 13,222 km^2^, all other Australian orchids = 5,256 km^2^). In contrast, *Sarcochilus* is neither widespread nor speciose (21 spp., 2,066 records, 5,038 km^2^ mean area of occupancy). It may be collected more often because it has a very attractive floral display and is used horticulturally, leading to stronger interest in collecting the wild species. Undercollection appears to be associated with taxonomic issues and/or restricted ranges with high levels of endemism. *Paracaleana* (14 spp., 2,700 km^2^) is somewhat taxonomically controversial with 11 endemic species restricted to the SWAFR (Hopper & Brown, 2006; Miller & Clements, 2014). *Dockrillia* (18 spp., 2,466 km^2^) is highly controversial with some molecular studies supporting splitting and others recommending reincorporating it into the genus *Dendrobium* (Burke, Bayly, Adams, & Ladiges, 2008; Schuiteman & Adams, 2011; cf. Clements, 2003). *Habenaria* (21 spp., 1,157 km^2^) is not taxonomically controversial, but the Australian species are mostly endemic and restricted to the tropics (Jones, 2006).

Our mapping confirms that Australian orchids are limited to 13% of the landmass, with obvious hotspots of higher diversity. In theory, there should be few barriers to orchid dispersal and colonization as their tiny, dust‐like seeds are readily transportable via wind and water, and vegetative reproduction allows new populations to arise from very few individual colonists (Arditti & Ghani, 2000; McCormick & Jacquemyn, 2014). However, experiments and molecular data reveal that orchids rarely achieve long‐range dispersal, and most dispersal events are over meters rather than kilometers (Brundrett, 2007; Givnish et al., 2016; Trapnell & Hamrick, 2005). Globally, orchid distribution is patchy with some smaller regions achieving much higher diversity than neighboring areas (e.g., Madagascar ~1,000 species vs. rest of Africa ~2,350 species; Mexico = 1,560 spp. vs. rest of North America = 394 spp.; Krupnick et al., 2013; WCSP, 2016). We identified three key Australian orchid diversity hotspots: the renowned SWAFR of Western Australia, plus the less well‐recognized east coast Victoria and New South Wales region, and northwestern and central Tasmania region. The SWAFR is a globally recognized biodiversity hotspot, ranked one of the world's top 25 priorities for conservation (Hopper & Gioia, 2004; Myers, Mittermeier, Mittermeier, Fonseca, & Kent, 2000). The orchids of the SWAFR have received considerable research attention addressing species interactions, rarity, biogeography, and conservation (Phillips et al., 2010; Phillips, Backhouse, Brown, & Hopper, 2009; Phillips, Brown, Dixon, & Hopper, 2007; Swarts & Dixon, 2009b). The orchids of Victoria, New South Wales, and Tasmania are yet to receive such wholistic, region‐based research.

We found that 211 orchid species (and presumably, their associated plant, fungal, and insect partners) are absent from Australian protected areas. While orchids in protected areas can be vulnerable to illegal collecting (especially in East Asia; Wraith & Pickering, 2017), protected areas may still offer better opportunities for policing this, and other major threats such as land clearing (Reiter et al., 2016). Species found only outside protected areas include 163 species from the five of the largest orchid genera (46 out of the 279 *Caladenia* species, 41/208 *Pterostylis*, 38/164 *Prasophyllum*, 13/110 *Thelymitra*, and 25/96 *Diuris*). Three of the species we found only outside protected areas are in the IUCN redlist (IUCN, 2018). A further 33 species are listed as threatened in the Australian Environment Protection and Biodiversity Conservation Act 1999 (SPRAT Database, 2018). However, these distributions and listings should be analyzed in detail given the possibility of taxonomic disagreement between these sources and the data we extracted from herbarium records.

Australian orchid diversity is the greatest within three of the six available biomes, but orchids are almost entirely absent from desert regions, unlike the other most diverse Australian angiosperm families (Fabaceae and Myrtaceae; Crisp, Cook, & Steane, 2004). Orchids appear to be particularly vulnerable to water availability; the absence of orchids from drier zones that we report here is consistent with the few available studies of orchid distribution and climate. For example, the distributions of *Pterostylis* orchid species and orchid‐associated mycorrhizal fungi in Australia are generally associated with water availability, including rainfall, drainage, and moisture index (Janes et al., 2010; McCormick & Jacquemyn, 2014; Nurfadilah et al., 2013). Chinese orchid distributions were also largely determined by net primary productivity and moisture index (Zhang et al., 2015). A study on the distribution of Macedonian orchids did not explicitly test for an effect of water availability, but the restricted distribution of wet meadow and bog specialist species was noted (Tsiftsis et al., 2008).

It is unclear whether it is the orchids or their mycorrhizae that are most dependent on water availability. Physiological constraints on orchids likely play a role in limiting the distribution of orchids in desert environments. Terrestrial orchids occur close to the soil surface which, in desert regions, can reach prohibitively high temperatures for maintaining adequate water balance and photosynthesis (Noy‐Meir, 1973). Orchids exhibit a wide range of leaf strategies including having both high and low specific leaf area across genera (SLA; a measure of the area invested, per unit carbon, in deploying photosynthetic leaf surfaces; Wright, Reich, Westoby, & Ackerly, 2004). High SLA is associated with acquisitive ecological strategies where leaf tissue is rapidly turned over to meet the energy requirements of growth and reproduction. This leaf strategy could be maintained in arid, desert environments—where water is limited—through ephemeral life histories, including rapid deployment of leaves and completion of the life cycle following rain, followed by energy conservation in tuberous rhizomes in dry periods (Noy‐Meir, 1973). Orchid flowering, seed set, and even pollination are sometimes triggered by rainfall events (Bodley, Beggs, Toft, & Gaskett, 2016; Brown & York, 2017; Fan et al., 2012). Some orchid species exhibit leaf trait adaptations compatible with drought conditions (e.g., sunken stoma, thick surface cuticles in Slipper Orchids, *Paphiopedilum*; Guan, Zhang, Guan, Li, & Hu, 2011) and so their paucity in Australian deserts may not be due to a lack of adaptive potential for arid environments. There may well be more orchid species yet to be formally collected from drier regions, for example, entirely subterranean and therefore rarely encountered species of *Rhizanthella,* although this genus is associated with Mediterranean rather than desert climates (Bougoure et al., 2008). Desert biomes are also unlikely to be suitable for maintaining mycorrhizal fungal due to low soil moisture content (McCormick & Jacquemyn, 2014; Nurfadilah et al., 2013).

Our data suggest that orchid speciation is facilitated by geographically linked or spatial factors, rather than any innate traits associated with particular orchid genera. We found that spatial patterns of orchid diversity were consistent at the level of genus and species, that is, regions with more genera also had more species. Furthermore, for the most diverse genera, species richness was the greatest in geographic regions of high general orchid diversity. Intriguingly, diversity was not associated with having a wider range size, or a broader tolerance for rainfall and temperature conditions, suggesting genera are not diversifying in response to invading new sites and adapting to their abiotic conditions. Therefore, the most important drivers of orchid speciation are likely to be both spatially linked and biotic. A critical factor is likely to be insect diversity; switching to a new pollinator species is instrumental in orchid speciation (Breitkopf, Onstein, Cafasso, Schlüter, & Cozzolino, 2015; Peakall & Whitehead, 2014; Peter & Johnson, 2014; Sun, Schlüter, Gross, & Schiestl, 2015). Mycorrhizal fungi diversity is less important than pollinators in orchid speciation (Phillips et al., 2014), but mycorrhizae do nonetheless determine the subset of sites that an orchid can colonize within its possible climatic range (McCormick & Jacquemyn, 2014; Nurfadilah et al., 2013). Dependence on mycorrhizae could underly the narrower range sizes we report here for orchids versus nonorchid species. Regions with diverse and abundant mycorrhizae may well support a richer orchid biota, although this is untested. Worldwide, orchid diversity hotspots do tend to occur in areas of high plant diversity (Cribb et al., 2003; Myers et al., 2000; Zhang et al., 2015), although no formal analyses have been performed.

Although several studies propose that orchid diversification is facilitated by their unique deceptive pollination systems (Givnish et al., 2015), here, we note no relationship between any particular pollination system and current species diversity. The most speciose genera in our study differ in their reproductive strategies: *Caladenia* has frequent transitions between food and sexual deception and rewarding strategies,* Pterostylis* are all sexually deceptive, *Diuris* has both food deceptive and rewarding species, whereas *Prasophyllum* species are all rewarding (Bates, 1984; Peakall & Beattie, 1991; Phillips et al., 2013; Phillips, Faast, Bower, Brown, & Peakall, 2009). Interestingly, this genus without any deceptive species, *Prasophyllum*, also had a significantly narrower range size. How abiotic and biotic factors influence the distribution of these pollination strategies on a landscape scale is an intriguing but untested avenue for future consideration (Herberstein, Baldwin, & Gaskett, 2014).

## AUTHOR CONTRIBUTIONS

ACG and RVG conceived the ideas, collated, and analyzed the data and wrote the manuscript.

## DATA ACCESSIBILITY

All data were extracted from publicly available online repositories. Details and URLs are provided in the Section Location.
