# Supplementary material for: Orchid diversity: Spatial and climatic patterns from herbarium records
Source: Ecol Evol. 2018 Oct 30;8(22):11235–45. doi: 10.1002/ece3.4598 (PMC6262934; doi:10.1002/ece3.4598)
Supplement: Supplementary file 1 [file ECE3-8-11235-s001.docx]

*Journal of Biogeography*

**APPENDICES**

**Orchid diversity: spatial and climatic patterns from Australian herbarium records**

A.C. Gaskett and R.V. Gallagher

**Appendix S1.1:** Orchid species extracted from Australia’s Virtual Herbarium and included in distribution analyses (n = 1538). Coverage in the protected area network was obtained by overlaying occurrence records with the 2014 Collaborative Australian Protected Area Database (<http://www.environment.gov.au/land/nrs/science/capad>) and extracting the number of species in each protected area.

| Species | Distribution | | | |
| --- | --- | --- | --- | --- |
|  | Absent from all protected areas  (n = 211) | Present only in protected areas  (n = 124) | | In protected & unprotected areas  (n=1203) |
| *Acianthus amplexicaulis* |  | |  | X |
| *Acianthus apprimus* |  | |  | X |
| *Acianthus borealis* |  | |  | X |
| *Acianthus caudatus* |  | |  | X |
| *Acianthus collinus* |  | |  | X |
| *Acianthus exiguus* |  | |  | X |
| *Acianthus exsertus* |  | |  | X |
| *Acianthus fornicatus* |  | |  | X |
| *Acianthus huegelii* | X | |  |  |
| *Acianthus pusillus* |  | |  | X |
| *Acianthus reniformis* |  | |  | X |
| *Acianthus* sp. aff. *fornicatus* (New England) |  | |  | X |
| *Acianthus sublestus* |  | |  | X |
| *Acianthus tenuissimus* |  | |  | X |
| *Acianthus viridis* |  | | X |  |
| *Acriopsis emarginata* |  | |  | X |
| *Acriopsis javanica* |  | |  | X |
| *Adenochilus nortonii* |  | |  | X |
| *Anoectochilus yatesiae* |  | |  | X |
| *Aphyllorchis anomala* |  | |  | X |
| *Aphyllorchis queenslandica* |  | |  | X |
| *Apostasia stylidioides* |  | |  | X |
| *Apostasia wallichii* |  | |  | X |
| *Appendicula australiensis* |  | |  | X |
| *Arthrochilus apectus* |  | | X |  |
| *Arthrochilus aquilus* |  | | X |  |
| *Arthrochilus byrnesii* |  | |  | X |
| *Arthrochilus corinnae* |  | | X |  |
| *Arthrochilus dockrillii* |  | |  | X |
| *Arthrochilus huntiana* |  | |  | X |
| *Arthrochilus huntianus* |  | |  | X |
| *Arthrochilus irritabilis* |  | |  | X |
| *Arthrochilus latipes* |  | |  | X |
| *Arthrochilus oreophilus* |  | |  | X |
| *Arthrochilus prolixus* |  | |  | X |
| *Arthrochilus rosulatus* |  | |  | X |
| *Arthrochilus sabulosus* |  | |  | X |
| *Arthrochilus stenophyllus* |  | | X |  |
| *Bromheadia pulchra* |  | |  | X |
| *Bromheadia venusta* |  | | X |  |
| *Bulbophyllum argyropus* |  | |  | X |
| *Bulbophyllum aurantiacum* |  | | X |  |
| *Bulbophyllum baileyi* |  | |  | X |
| *Bulbophyllum boonjee* |  | | X |  |
| *Bulbophyllum bracteatum* |  | |  | X |
| *Bulbophyllum caldericola* |  | | X |  |
| *Bulbophyllum cilioglossum* | X | |  |  |
| *Bulbophyllum crassulifolium* |  | |  | X |
| *Bulbophyllum elisae* |  | |  | X |
| *Bulbophyllum evasum* |  | |  | X |
| *Bulbophyllum exiguum* |  | |  | X |
| *Bulbophyllum fruticicola* |  | | X |  |
| *Bulbophyllum gadgarrense* |  | |  | X |
| *Bulbophyllum globuliforme* |  | |  | X |
| *Bulbophyllum gracillimum* |  | | X |  |
| *Bulbophyllum grandimesense* |  | | X |  |
| *Bulbophyllum intermedium* |  | | X |  |
| *Bulbophyllum johnsonii* |  | |  | X |
| *Bulbophyllum lageniforme* |  | |  | X |
| *Bulbophyllum lamingtonense* |  | | X |  |
| *Bulbophyllum lewisense* |  | | X |  |
| *Bulbophyllum lilianiae* |  | |  | X |
| *Bulbophyllum longiflorum* |  | |  | X |
| *Bulbophyllum macphersonii* |  | |  | X |
| *Bulbophyllum masdevalliaceum* |  | |  | X |
| *Bulbophyllum minutissimum* |  | |  | X |
| *Bulbophyllum nematopodum* |  | |  | X |
| *Bulbophyllum newportii* |  | |  | X |
| *Bulbophyllum prenticei* | X | |  |  |
| *Bulbophyllum radicans* |  | |  | X |
| *Bulbophyllum revolutum* |  | | X |  |
| *Bulbophyllum schillerianum* |  | |  | X |
| *Bulbophyllum shepherdii* |  | |  | X |
| *Bulbophyllum sladeanum* |  | |  | X |
| *Bulbophyllum wadsworthii* |  | |  | X |
| *Bulbophyllum weinthalii* |  | |  | X |
| *Bulbophyllum whitei* |  | | X |  |
| *Bulbophyllum windsorense* |  | | X |  |
| *Bulbophyllum wolfei* |  | | X |  |
| *Burnettia cuneata* |  | |  | X |
| *Cadetia clausa* |  | |  | X |
| *Cadetia collinsii* |  | | X |  |
| *Cadetia hispida* | X | |  |  |
| *Cadetia maideniana* |  | |  | X |
| *Cadetia taylori* |  | |  | X |
| *Cadetia uniflos* |  | |  | X |
| *Cadetia wariana* |  | |  | X |
| *Caladenia abbreviata* |  | |  | X |
| *Caladenia actensis* |  | |  | X |
| *Caladenia aestiva* |  | |  | X |
| *Caladenia alata* |  | |  | X |
| *Caladenia alba* |  | |  | X |
| *Caladenia alpina* |  | |  | X |
| *Caladenia amnicola* | X | |  |  |
| *Caladenia amoena* | X | |  |  |
| *Caladenia ampla* |  | |  | X |
| *Caladenia amplexans* |  | |  | X |
| *Caladenia ancylosa* | X | |  |  |
| *Caladenia angustata* |  | |  | X |
| *Caladenia anthracina* |  | |  | X |
| *Caladenia aphylla* |  | |  | X |
| *Caladenia applanata* | X | |  |  |
| *Caladenia arenaria* |  | |  | X |
| *Caladenia arenicola* |  | |  | X |
| *Caladenia argocalla* |  | |  | X |
| *Caladenia arrecta* |  | |  | X |
| *Caladenia atrata* |  | |  | X |
| *Caladenia atrochila* |  | |  | X |
| *Caladenia atroclavia* |  | |  | X |
| *Caladenia atrovespa* |  | | X |  |
| *Caladenia attingens* | X | |  |  |
| *Caladenia audasii* |  | |  | X |
| *Caladenia aurantiaca* |  | |  | X |
| *Caladenia aurulenta* |  | |  | X |
| *Caladenia australis* |  | |  | X |
| *Caladenia barbarella* |  | |  | X |
| *Caladenia barbarossa* |  | |  | X |
| *Caladenia behrii* |  | |  | X |
| *Caladenia bicalliata* |  | |  | X |
| *Caladenia bicolor* | X | |  |  |
| *Caladenia brachyscapa* |  | |  | X |
| *Caladenia branwhitei* | X | |  |  |
| *Caladenia brevisura* |  | |  | X |
| *Caladenia brownii* |  | |  | X |
| *Caladenia brumalis* |  | |  | X |
| *Caladenia bryceana* |  | |  | X |
| *Caladenia busselliana* | X | |  |  |
| *Caladenia caerulea* |  | |  | X |
| *Caladenia caesarea* |  | |  | X |
| *Caladenia cairnsiana* |  | |  | X |
| *Caladenia calcicola* |  | |  | X |
| *Caladenia callitrophila* | X | |  |  |
| *Caladenia campbellii* | X | |  |  |
| *Caladenia capillata* |  | |  | X |
| *Caladenia carnea* |  | |  | X |
| *Caladenia catenata* |  | |  | X |
| *Caladenia caudata* |  | |  | X |
| *Caladenia chamaephylla* |  | | X |  |
| *Caladenia chapmanii* |  | |  | X |
| *Caladenia christineae* |  | |  | X |
| *Caladenia citrina* |  | |  | X |
| *Caladenia clarkiae* |  | |  | X |
| *Caladenia clavigera* |  | |  | X |
| *Caladenia clavula* |  | |  | X |
| *Caladenia cleistantha* |  | |  | X |
| *Caladenia coactilis* |  | |  | X |
| *Caladenia colorata* |  | |  | X |
| *Caladenia concolor* |  | |  | X |
| *Caladenia conferta* |  | |  | X |
| *Caladenia congesta* |  | |  | X |
| *Caladenia corynephora* |  | |  | X |
| *Caladenia cracens* |  | |  | X |
| *Caladenia crebra* |  | |  | X |
| *Caladenia cremna* | X | |  |  |
| *Caladenia cretacea* |  | |  | X |
| *Caladenia cristata* |  | |  | X |
| *Caladenia cruciformis* |  | |  | X |
| *Caladenia cruscula* |  | |  | X |
| *Caladenia cucullata* |  | |  | X |
| *Caladenia curtisepala* |  | |  | X |
| *Caladenia decora* |  | |  | X |
| *Caladenia deformis* |  | |  | X |
| *Caladenia denticulata* |  | |  | X |
| *Caladenia dienema* |  | |  | X |
| *Caladenia dimidia* |  | |  | X |
| *Caladenia dimorpha* |  | |  | X |
| *Caladenia discoidea* |  | |  | X |
| *Caladenia dorrienii* |  | |  | X |
| *Caladenia douglasiorum* |  | | X |  |
| *Caladenia doutchae* |  | |  | X |
| *Caladenia doutchiae* |  | |  | X |
| *Caladenia drakeoides* | X | |  |  |
| *Caladenia drummondii* |  | |  | X |
| *Caladenia dundasiae* | X | |  |  |
| *Caladenia echidnachila* |  | |  | X |
| *Caladenia elegans* | X | |  |  |
| *Caladenia elongata* |  | |  | X |
| *Caladenia eminens* | X | |  |  |
| *Caladenia ensata* |  | |  | X |
| *Caladenia ensigera* |  | | X |  |
| *Caladenia ericksoniae* |  | |  | X |
| *Caladenia erythrochila* | X | |  |  |
| *Caladenia evanescens* | X | |  |  |
| *Caladenia excelsa* |  | |  | X |
| *Caladenia exilis* | X | |  |  |
| *Caladenia exstans* |  | |  | X |
| *Caladenia falcata* |  | |  | X |
| *Caladenia ferruginea* |  | |  | X |
| *Caladenia filamentosa* |  | |  | X |
| *Caladenia filifera* |  | |  | X |
| *Caladenia fitzgeraldii* |  | |  | X |
| *Caladenia flaccida* |  | |  | X |
| *Caladenia flava* |  | |  | X |
| *Caladenia flavovirens* |  | |  | X |
| *Caladenia flindersica* |  | |  | X |
| *Caladenia floribunda* | X | |  |  |
| *Caladenia footeana* |  | |  | X |
| *Caladenia formosa* |  | |  | X |
| *Caladenia fragrantissima* |  | |  | X |
| *Caladenia fuliginosa* | X | |  |  |
| *Caladenia fulva* |  | |  | X |
| *Caladenia fuscata* |  | |  | X |
| *Caladenia fuscolutescens* |  | |  | X |
| *Caladenia gardneri* |  | |  | X |
| *Caladenia gemmata* |  | |  | X |
| *Caladenia georgei* |  | |  | X |
| *Caladenia gladiolata* |  | |  | X |
| *Caladenia gracilis* |  | |  | X |
| *Caladenia gracillima* |  | |  | X |
| *Caladenia graminifolia* |  | |  | X |
| *Caladenia grampiana* |  | |  | X |
| *Caladenia graniticola* |  | |  | X |
| *Caladenia granitora* |  | |  | X |
| *Caladenia harringtoniae* |  | |  | X |
| *Caladenia hastata* | X | |  |  |
| *Caladenia heberleana* |  | |  | X |
| *Caladenia helvina* |  | |  | X |
| *Caladenia hiemalis* |  | |  | X |
| *Caladenia hildae* |  | |  | X |
| *Caladenia hillmanii* |  | |  | X |
| *Caladenia hirta* |  | |  | X |
| *Caladenia hoffmanii* |  | |  | X |
| *Caladenia horistes* |  | |  | X |
| *Caladenia huegelii* |  | |  | X |
| *Caladenia incensa* |  | |  | X |
| *Caladenia incrassata* |  | |  | X |
| *Caladenia infundibularis* |  | |  | X |
| *Caladenia insularis* |  | |  | X |
| *Caladenia integra* |  | |  | X |
| *Caladenia interanea* |  | | X |  |
| *Caladenia interjacens* |  | |  | X |
| *Caladenia intuta* | X | |  |  |
| *Caladenia iridescens* |  | |  | X |
| *Caladenia ixioides* |  | | X |  |
| *Caladenia latifolia* |  | |  | X |
| *Caladenia leptochila* |  | |  | X |
| *Caladenia leptoclavia* | X | |  |  |
| *Caladenia lindleyana* |  | |  | X |
| *Caladenia lobata* |  | |  | X |
| *Caladenia lodgeana* |  | |  | X |
| *Caladenia longicauda* |  | |  | X |
| *Caladenia longiclavata* |  | |  | X |
| *Caladenia longifimbriata* | X | |  |  |
| *Caladenia longii* | X | |  |  |
| *Caladenia lorea* |  | |  | X |
| *Caladenia lowanensis* |  | |  | X |
| *Caladenia luteola* | X | |  |  |
| *Caladenia lyallii* |  | |  | X |
| *Caladenia macroclavia* |  | |  | X |
| *Caladenia macrostylis* |  | |  | X |
| *Caladenia magniclavata* |  | |  | X |
| *Caladenia magnifica* |  | |  | X |
| *Caladenia marginata* |  | |  | X |
| *Caladenia maritima* |  | |  | X |
| *Caladenia melanema* |  | |  | X |
| *Caladenia mentiens* |  | |  | X |
| *Caladenia menziesii* |  | |  | X |
| *Caladenia meridionalis* |  | |  | X |
| *Caladenia mesocera* |  | |  | X |
| *Caladenia microchila* |  | |  | X |
| *Caladenia minor* |  | |  | X |
| *Caladenia montana* |  | |  | X |
| *Caladenia moschata* |  | | X |  |
| *Caladenia multiclavia* |  | |  | X |
| *Caladenia nana* |  | |  | X |
| *Caladenia necrophylla* |  | |  | X |
| *Caladenia nivalis* |  | | X |  |
| *Caladenia nobilis* |  | |  | X |
| *Caladenia occidentalis* |  | |  | X |
| *Caladenia oenochila* |  | |  | X |
| *Caladenia oreophila* | X | |  |  |
| *Caladenia orientalis* |  | |  | X |
| *Caladenia ornata* |  | |  | X |
| *Caladenia ovata* |  | |  | X |
| *Caladenia pachychila* |  | |  | X |
| *Caladenia pallida* |  | |  | X |
| *Caladenia paludosa* |  | |  | X |
| *Caladenia paradoxa* |  | |  | X |
| *Caladenia parva* |  | |  | X |
| *Caladenia pectinata* |  | |  | X |
| *Caladenia peisleyi* | X | |  |  |
| *Caladenia pendens* | X | |  |  |
| *Caladenia petrensis* |  | |  | X |
| *Caladenia phaeoclavia* |  | |  | X |
| *Caladenia pholcoidea* |  | |  | X |
| *Caladenia picta* |  | |  | X |
| *Caladenia pilotensis* |  | | X |  |
| *Caladenia plicata* |  | |  | X |
| *Caladenia polychroma* |  | |  | X |
| *Caladenia postea* |  | |  | X |
| *Caladenia praecox* |  | |  | X |
| *Caladenia procera* | X | |  |  |
| *Caladenia prolata* |  | |  | X |
| *Caladenia pulchra* |  | |  | X |
| *Caladenia pumila* | X | |  |  |
| *Caladenia pusilla* |  | |  | X |
| *Caladenia quadrifaria* |  | |  | X |
| *Caladenia radialis* |  | |  | X |
| *Caladenia radiata* |  | |  | X |
| *Caladenia remota* | X | |  |  |
| *Caladenia reniformis* | X | |  |  |
| *Caladenia reptans* |  | |  | X |
| *Caladenia reticulata* |  | |  | X |
| *Caladenia rhomboidiformis* |  | |  | X |
| *Caladenia richardsiorum* |  | |  | X |
| *Caladenia rigens* | X | |  |  |
| *Caladenia rigida* |  | |  | X |
| *Caladenia rileyi* | X | |  |  |
| *Caladenia robinsonii* |  | |  | X |
| *Caladenia roei* |  | |  | X |
| *Caladenia rosella* | X | |  |  |
| *Caladenia saccharata* |  | |  | X |
| *Caladenia saggicola* | X | |  |  |
| *Caladenia sanguinea* |  | |  | X |
| *Caladenia saxatilis* |  | |  | X |
| *Caladenia saxicola* |  | |  | X |
| *Caladenia septuosa* |  | |  | X |
| *Caladenia sericea* |  | |  | X |
| *Caladenia serotina* |  | |  | X |
| *Caladenia serrata* | X | |  |  |
| *Caladenia sigmoidea* |  | |  | X |
| *Caladenia simulans* | X | |  |  |
| *Caladenia* sp. A |  | |  | X |
| *Caladenia* sp. aff. *fitzgeraldii* (Mallacoota) | X | |  |  |
| *Caladenia* sp. Bordertown (R.S.Rogers 788) |  | |  | X |
| *Caladenia* sp. Kilcoy Creek (R.Crane 1286) |  | |  | X |
| *Caladenia* sp. Rothsay (G.Brockman GBB404) | X | |  |  |
| *Caladenia speciosa* |  | |  | X |
| *Caladenia splendens* |  | |  | X |
| *Caladenia starteorum* |  | |  | X |
| *Caladenia stellata* |  | |  | X |
| *Caladenia stricta* |  | |  | X |
| *Caladenia strigosa* |  | | X |  |
| *Caladenia subtilis* | X | |  |  |
| *Caladenia sylvicola* | X | |  |  |
| *Caladenia tensa* |  | |  | X |
| *Caladenia tentaculata* |  | |  | X |
| *Caladenia tessellata* |  | |  | X |
| *Caladenia testacea* |  | |  | X |
| *Caladenia thinicola* |  | |  | X |
| *Caladenia thysanochila* | X | |  |  |
| *Caladenia tonellii* |  | |  | X |
| *Caladenia transitoria* |  | |  | X |
| *Caladenia triangularis* |  | |  | X |
| *Caladenia uliginosa* |  | |  | X |
| *Caladenia ultima* |  | |  | X |
| *Caladenia unita* |  | |  | X |
| *Caladenia ustulata* |  | |  | X |
| *Caladenia valida* |  | |  | X |
| *Caladenia variegata* | X | |  |  |
| *Caladenia venusta* |  | |  | X |
| *Caladenia verrucosa* |  | |  | X |
| *Caladenia versicolor* |  | |  | X |
| *Caladenia villosissima* |  | | X |  |
| *Caladenia viridescens* |  | |  | X |
| *Caladenia voigtii* |  | |  | X |
| *Caladenia vulgaris* |  | |  | X |
| *Caladenia vulgata* |  | |  | X |
| *Caladenia wanosa* |  | |  | X |
| *Caladenia williamsiae* | X | |  |  |
| *Caladenia winfieldii* |  | |  | X |
| *Caladenia woolcockiorum* |  | |  | X |
| *Caladenia xantha* |  | |  | X |
| *Caladenia xanthochila* |  | |  | X |
| *Caladenia xantholeuca* |  | |  | X |
| *Caladenia zephyra* |  | |  | X |
| *Calanthe australasica* |  | |  | X |
| *Calanthe triplicata* |  | |  | X |
| *Calanthe veratrifolia* |  | | X |  |
| *Caleana major* |  | |  | X |
| *Caleana minor* |  | |  | X |
| *Caleana nigrita* |  | |  | X |
| *Caleana nublingii* |  | | X |  |
| *Calochilus ammobius* | X | |  |  |
| *Calochilus caeruleus* |  | |  | X |
| *Calochilus caesius* |  | |  | X |
| *Calochilus campestris* |  | |  | X |
| *Calochilus cleistanthus* |  | | X |  |
| *Calochilus cupreus* | X | |  |  |
| *Calochilus gracillimus* |  | |  | X |
| *Calochilus grandiflorus* |  | |  | X |
| *Calochilus herbaceus* |  | |  | X |
| *Calochilus holtzei* |  | |  | X |
| *Calochilus imberbis* |  | |  | X |
| *Calochilus imperiosus* |  | |  | X |
| *Calochilus kalaru* | X | |  |  |
| *Calochilus metallicus* | X | |  |  |
| *Calochilus montanus* |  | |  | X |
| *Calochilus paludosus* |  | |  | X |
| *Calochilus platychilus* |  | |  | X |
| *Calochilus praeltus* |  | | X |  |
| *Calochilus pruinosus* |  | |  | X |
| *Calochilus psednus* |  | | X |  |
| *Calochilus pulchellus* |  | |  | X |
| *Calochilus richiae* | X | |  |  |
| *Calochilus robertsoni* |  | |  | X |
| *Calochilus robertsonii* |  | |  | X |
| *Calochilus russeus* |  | |  | X |
| *Calochilus sandrae* |  | |  | X |
| *Calochilus saprophyticus* |  | |  | X |
| *Calochilus stramenicola* |  | |  | X |
| *Calochilus therophilus* |  | |  | X |
| *Calochilus uliginosus* |  | |  | X |
| *Cheirostylis notialis* |  | |  | X |
| *Cheirostylis ovata* |  | |  | X |
| *Chiloglottis anaticeps* |  | |  | X |
| *Chiloglottis chlorantha* |  | |  | X |
| *Chiloglottis cornuta* |  | |  | X |
| *Chiloglottis diphylla* |  | |  | X |
| *Chiloglottis dockrillii* |  | | X |  |
| *Chiloglottis formicifera* |  | |  | X |
| *Chiloglottis grammata* |  | |  | X |
| *Chiloglottis gunnii* |  | |  | X |
| *Chiloglottis jeanesii* |  | |  | X |
| *Chiloglottis longiclavata* |  | |  | X |
| *Chiloglottis palachila* |  | |  | X |
| *Chiloglottis platychila* |  | |  | X |
| *Chiloglottis platyptera* |  | |  | X |
| *Chiloglottis pluricallata* |  | |  | X |
| *Chiloglottis reflexa* |  | |  | X |
| *Chiloglottis seminuda* |  | |  | X |
| *Chiloglottis* sp. A |  | | X |  |
| *Chiloglottis* sp. aff. *chlorantha* (Gippsland) | X | |  |  |
| *Chiloglottis* sp. aff. *formicifera* (Bald Rock) |  | |  | X |
| *Chiloglottis* sp. aff. *palachila* (Bathurst) | X | |  |  |
| *Chiloglottis* sp. aff. *pluricallata* (Northern Tablelands) |  | | X |  |
| *Chiloglottis sphaerula* |  | |  | X |
| *Chiloglottis sphyrnoides* |  | |  | X |
| *Chiloglottis sylvestris* |  | |  | X |
| *Chiloglottis trapeziformis* |  | |  | X |
| *Chiloglottis triceratops* |  | |  | X |
| *Chiloglottis trilabra* |  | |  | X |
| *Chiloglottis trullata* |  | | X |  |
| *Chiloglottis truncata* |  | |  | X |
| *Chiloglottis turfosa* |  | |  | X |
| *Chiloglottis valida* |  | |  | X |
| *Chiloschista phyllorhiza* |  | |  | X |
| *Cooktownia robertsii* |  | | X |  |
| *Corunastylis acuminata* |  | |  | X |
| *Corunastylis alticola* |  | |  | X |
| *Corunastylis anthracina* |  | |  | X |
| *Corunastylis apostasioides* |  | |  | X |
| *Corunastylis archeri* |  | |  | X |
| *Corunastylis arrecta* |  | |  | X |
| *Corunastylis bishopii* |  | | X |  |
| *Corunastylis ciliata* |  | |  | X |
| *Corunastylis citriodora* | X | |  |  |
| *Corunastylis clivicola* |  | |  | X |
| *Corunastylis conferta* |  | |  | X |
| *Corunastylis cornuta* |  | |  | X |
| *Corunastylis cranei* |  | |  | X |
| *Corunastylis densa* |  | |  | X |
| *Corunastylis despectans* |  | |  | X |
| *Corunastylis ectopa* |  | | X |  |
| *Corunastylis eriochila* |  | |  | X |
| *Corunastylis filiformis* |  | |  | X |
| *Corunastylis fimbriata* |  | |  | X |
| *Corunastylis formosa* |  | |  | X |
| *Corunastylis laminata* |  | |  | X |
| *Corunastylis littoralis* | X | |  |  |
| *Corunastylis morina* |  | | X |  |
| *Corunastylis morrisii* |  | |  | X |
| *Corunastylis nigricans* |  | |  | X |
| *Corunastylis nuda* |  | |  | X |
| *Corunastylis nudiscapa* |  | |  | X |
| *Corunastylis oligantha* |  | |  | X |
| *Corunastylis ostrina* |  | |  | X |
| *Corunastylis parvicalla* |  | | X |  |
| *Corunastylis psammophila* |  | | X |  |
| *Corunastylis pumila* |  | |  | X |
| *Corunastylis rufa* |  | |  | X |
| *Corunastylis ruppii* |  | |  | X |
| *Corunastylis sagittifera* |  | |  | X |
| *Corunastylis sigmoidea* | X | |  |  |
| *Corunastylis simulans* |  | |  | X |
| *Corunastylis superba* |  | |  | X |
| *Corunastylis systena* |  | | X |  |
| *Corunastylis tasmanica* |  | |  | X |
| *Corunastylis tepperi* |  | |  | X |
| *Corunastylis trifida* |  | |  | X |
| *Corunastylis turfosa* |  | | X |  |
| *Corunastylis woollsii* |  | |  | X |
| *Corybas abditus* |  | |  | X |
| *Corybas abellianus* |  | |  | X |
| *Corybas aconitiflorus* |  | |  | X |
| *Corybas barbarae* |  | |  | X |
| *Corybas cerasinus* |  | |  | X |
| *Corybas dentatus* | X | |  |  |
| *Corybas despectans* |  | |  | X |
| *Corybas diemenicus* |  | |  | X |
| *Corybas dilatatus* |  | |  | X |
| *Corybas dowlingii* |  | |  | X |
| *Corybas expansus* |  | |  | X |
| *Corybas fimbriatus* |  | |  | X |
| *Corybas fordhamii* |  | |  | X |
| *Corybas grumulus* |  | | X |  |
| *Corybas hispidus* |  | |  | X |
| *Corybas limpidus* |  | |  | X |
| *Corybas montanus* |  | |  | X |
| *Corybas neocaledonicus* |  | |  | X |
| *Corybas pruinosus* |  | |  | X |
| *Corybas recurvus* |  | |  | X |
| *Corybas* sp. aff. *dilatatus* |  | |  | X |
| *Corybas undulatus* |  | |  | X |
| *Corybas unguiculatus* |  | |  | X |
| *Corymborkis veratrifolia* |  | |  | X |
| *Corysanthes fimbriata* |  | |  | X |
| *Corysanthes pruinosa* |  | |  | X |
| *Crepidium flavovirens* |  | | X |  |
| *Crepidium fontinale* |  | |  | X |
| *Cryptostylis erecta* |  | |  | X |
| *Cryptostylis hunteriana* |  | |  | X |
| *Cryptostylis leptochila* |  | |  | X |
| *Cryptostylis ovata* |  | |  | X |
| *Cryptostylis subulata* |  | |  | X |
| *Cyanicula amplexans* |  | |  | X |
| *Cyanicula aperta* |  | |  | X |
| *Cyanicula ashbyae* |  | |  | X |
| *Cyanicula caerulea* |  | |  | X |
| *Cyanicula deformis* |  | |  | X |
| *Cyanicula fragrans* |  | |  | X |
| *Cyanicula gemmata* |  | |  | X |
| *Cyanicula gertrudiae* |  | |  | X |
| *Cyanicula ixioides* |  | |  | X |
| *Cyanicula nikulinskyae* |  | |  | X |
| *Cyanicula sericea* | X | |  |  |
| *Cymbidium canaliculatum* |  | |  | X |
| *Cymbidium iridifolium* |  | |  | X |
| *Cymbidium leroyi* |  | |  | X |
| *Cymbidium madidum* |  | | X |  |
| *Cymbidium suave* |  | |  | X |
| *Cyrtostylis huegelii* |  | |  | X |
| *Cyrtostylis reniformis* |  | |  | X |
| *Cyrtostylis robusta* |  | |  | X |
| *Cyrtostylis tenuissima* |  | |  | X |
| *Danhatchia australis* |  | |  | X |
| *Demorchis queenslandica* |  | |  | X |
| *Dendrobium adae* |  | | X |  |
| *Dendrobium aemulum* |  | |  | X |
| *Dendrobium affine* |  | |  | X |
| *Dendrobium agrostophyllum* |  | |  | X |
| *Dendrobium baileyi* |  | |  | X |
| *Dendrobium beckleri* |  | |  | X |
| *Dendrobium bifalce* |  | |  | X |
| *Dendrobium bigibbum* |  | |  | X |
| *Dendrobium bowmanii* |  | |  | X |
| *Dendrobium brevicaudum* |  | |  | X |
| *Dendrobium cacatua* |  | | X |  |
| *Dendrobium calamiforme* |  | |  | X |
| *Dendrobium canaliculatum* |  | |  | X |
| *Dendrobium capitisyork* |  | |  | X |
| *Dendrobium carrii* |  | |  | X |
| *Dendrobium cucumerinum* |  | |  | X |
| *Dendrobium curvicaule* |  | |  | X |
| *Dendrobium delicatum* |  | |  | X |
| *Dendrobium discolor* |  | |  | X |
| *Dendrobium dolichophyllum* |  | |  | X |
| *Dendrobium fairfaxii* |  | |  | X |
| *Dendrobium falcorostrum* |  | |  | X |
| *Dendrobium fellowsii* |  | |  | X |
| *Dendrobium finniganensis* |  | |  | X |
| *Dendrobium fleckeri* |  | | X |  |
| *Dendrobium gracilicaule* |  | |  | X |
| *Dendrobium gracillimum* |  | |  | X |
| *Dendrobium jonesii* |  | |  | X |
| *Dendrobium kestevenii* |  | |  | X |
| *Dendrobium kingianum* | X | |  |  |
| *Dendrobium lichenastrum* |  | |  | X |
| *Dendrobium linguiforme* |  | |  | X |
| *Dendrobium lobbii* |  | |  | X |
| *Dendrobium luteocilium* |  | |  | X |
| *Dendrobium malbrownii* |  | |  | X |
| *Dendrobium masonii* |  | |  | X |
| *Dendrobium melaleucaphilum* |  | | X |  |
| *Dendrobium monophyllum* |  | |  | X |
| *Dendrobium mortii* |  | |  | X |
| *Dendrobium nugentii* |  | |  | X |
| *Dendrobium pedunculatum* |  | |  | X |
| *Dendrobium prenticei* | X | |  |  |
| *Dendrobium pugioniforme* |  | |  | X |
| *Dendrobium racemosum* |  | |  | X |
| *Dendrobium rex* |  | |  | X |
| *Dendrobium rigidum* |  | |  | X |
| *Dendrobium ruppianum* |  | |  | X |
| *Dendrobium schneiderae* |  | |  | X |
| *Dendrobium schoeninum* |  | |  | X |
| *Dendrobium smillieae* |  | |  | X |
| *Dendrobium speciosum* |  | |  | X |
| *Dendrobium striolatum* |  | |  | X |
| *Dendrobium stuartii* |  | |  | X |
| *Dendrobium suffusum* |  | |  | X |
| *Dendrobium tarberi* | X | |  |  |
| *Dendrobium tenuissimum* |  | |  | X |
| *Dendrobium teretifolium* |  | |  | X |
| *Dendrobium tetragonum* |  | |  | X |
| *Dendrobium toressae* |  | |  | X |
| *Dendrobium trilamellatum* |  | |  | X |
| *Dendrobium wassellii* |  | |  | X |
| *Didymoplexis pallens* |  | | X |  |
| *Dienia latifolia* |  | |  | X |
| *Dienia montana* |  | | X |  |
| *Dienia ophrydis* |  | |  | X |
| *Diplocaulobium glabrum* |  | |  | X |
| *Dipodium atropurpureum* |  | |  | X |
| *Dipodium campanulatum* |  | |  | X |
| *Dipodium elegantulum* |  | |  | X |
| *Dipodium ensifolium* |  | |  | X |
| *Dipodium hamiltonianum* |  | |  | X |
| *Dipodium pardalinum* |  | |  | X |
| *Dipodium pictum* |  | |  | X |
| *Dipodium pulchellum* |  | |  | X |
| *Dipodium punctatum* |  | |  | X |
| *Dipodium roseum* |  | |  | X |
| *Dipodium stenocheilum* |  | |  | X |
| *Dipodium variegatum* |  | |  | X |
| *Diteilis petricola* |  | |  | X |
| *Diteilis simmondsii* |  | | X |  |
| *Diuris abbreviata* |  | |  | X |
| *Diuris aequalis* |  | |  | X |
| *Diuris* aff. *amplissima* |  | |  | X |
| *Diuris* aff. *corymbosa* |  | | X |  |
| *Diuris alba* | X | |  |  |
| *Diuris althoferi* |  | |  | X |
| *Diuris amplissima* |  | |  | X |
| *Diuris arenaria* |  | |  | X |
| *Diuris aurea* |  | |  | X |
| *Diuris basaltica* |  | |  | X |
| *Diuris behrii* | X | |  |  |
| *Diuris bracteata* |  | |  | X |
| *Diuris brevifolia* |  | |  | X |
| *Diuris brevissima* |  | |  | X |
| *Diuris brumalis* |  | |  | X |
| *Diuris byronensis* |  | |  | X |
| *Diuris callitrophila* | X | |  |  |
| *Diuris carinata* | X | |  |  |
| *Diuris chrysantha* |  | |  | X |
| *Diuris chryseopsis* |  | |  | X |
| *Diuris citrina* |  | |  | X |
| *Diuris concinna* | X | |  |  |
| *Diuris conspicillata* |  | |  | X |
| *Diuris corymbosa* | X | |  |  |
| *Diuris cuneata* |  | |  | X |
| *Diuris curta* |  | |  | X |
| *Diuris daltonii* |  | | X |  |
| *Diuris dendrobioides* | X | |  |  |
| *Diuris disposita* |  | |  | X |
| *Diuris drummondii* |  | |  | X |
| *Diuris eborensis* |  | |  | X |
| *Diuris eburnea* |  | |  | X |
| *Diuris elongata* | X | |  |  |
| *Diuris emarginata* | X | |  |  |
| *Diuris exitela* |  | |  | X |
| *Diuris fastidiosa* |  | |  | X |
| *Diuris filifolia* | X | |  |  |
| *Diuris flavescens* |  | |  | X |
| *Diuris flavopurpurea* |  | |  | X |
| *Diuris fragrantissima* | X | |  |  |
| *Diuris fucosa* | X | |  |  |
| *Diuris goonooensis* | X | |  |  |
| *Diuris gregaria* |  | |  | X |
| *Diuris heberlei* | X | |  |  |
| *Diuris immaculata* |  | |  | X |
| *Diuris laevis* | X | |  |  |
| *Diuris lanceolata* |  | |  | X |
| *Diuris laxiflora* |  | |  | X |
| *Diuris lineata* |  | |  | X |
| *Diuris longifolia* | X | |  |  |
| *Diuris luteola* |  | |  | X |
| *Diuris maculata* |  | |  | X |
| *Diuris maculosissima* |  | |  | X |
| *Diuris magnifica* | X | |  |  |
| *Diuris micrantha* |  | |  | X |
| *Diuris minor* |  | |  | X |
| *Diuris monticola* |  | |  | X |
| *Diuris nebulosa* |  | |  | X |
| *Diuris nigromontana* | X | |  |  |
| *Diuris ochroma* |  | |  | X |
| *Diuris oporina* |  | |  | X |
| *Diuris orientis* |  | |  | X |
| *Diuris palachila* |  | |  | X |
| *Diuris palustris* |  | |  | X |
| *Diuris pardina* |  | |  | X |
| *Diuris parvipetala* |  | |  | X |
| *Diuris pauciflora* |  | |  | X |
| *Diuris pedunculata* |  | |  | X |
| *Diuris perialla* |  | |  | X |
| *Diuris picta* | X | |  |  |
| *Diuris platichila* |  | |  | X |
| *Diuris polymorpha* |  | |  | X |
| *Diuris porrifolia* | X | |  |  |
| *Diuris praecox* |  | |  | X |
| *Diuris protena* |  | |  | X |
| *Diuris pulchella* |  | |  | X |
| *Diuris punctata* |  | |  | X |
| *Diuris purdiei* |  | |  | X |
| *Diuris recurva* |  | |  | X |
| *Diuris secundiflora* |  | |  | X |
| *Diuris semilunulata* | X | |  |  |
| *Diuris setacea* |  | |  | X |
| *Diuris* sp. aff. *alba* (New England Swamps) |  | |  | X |
| *Diuris* sp. aff. *alba* (Northern Tablelands) |  | |  | X |
| *Diuris* sp. aff. *chrysantha* (North Coast) | X | |  |  |
| *Diuris* sp. aff. *dendrobioides* (Hunter Valley) |  | |  | X |
| *Diuris* sp. aff. *dendrobioides* (Monaro) |  | |  | X |
| *Diuris* sp. aff. *ochroma* (New England) | X | |  |  |
| *Diuris* sp. aff. *punctata* (Colo River) |  | |  | X |
| *Diuris striata* |  | | X |  |
| *Diuris subalpina* | X | |  |  |
| *Diuris sulphurea* |  | |  | X |
| *Diuris systena* |  | |  | X |
| *Diuris tricolor* | X | |  |  |
| *Diuris unica* |  | |  | X |
| *Diuris venosa* |  | |  | X |
| *Dockrillia banksii* |  | |  | X |
| *Dockrillia bowmanii* |  | |  | X |
| *Dockrillia brevicauda* |  | |  | X |
| *Dockrillia calamiformis* |  | | X |  |
| *Dockrillia cucumerina* |  | |  | X |
| *Dockrillia dolichophylla* |  | |  | X |
| *Dockrillia fairfaxii* |  | |  | X |
| *Dockrillia linguiforme* |  | |  | X |
| *Dockrillia linguiformis* |  | |  | X |
| *Dockrillia mortii* |  | |  | X |
| *Dockrillia nugentii* |  | |  | X |
| *Dockrillia pugioniformis* |  | |  | X |
| *Dockrillia racemosa* |  | |  | X |
| *Dockrillia rigida* |  | |  | X |
| *Dockrillia schoenina* |  | |  | X |
| *Dockrillia striolata* |  | |  | X |
| *Dockrillia teretifolia* |  | |  | X |
| *Dockrillia wassellii* |  | |  | X |
| *Drakaea concolor* |  | |  | X |
| *Drakaea confluens* |  | |  | X |
| *Drakaea elastica* |  | |  | X |
| *Drakaea fitzgeraldii* | X | |  |  |
| *Drakaea glyptodon* | X | |  |  |
| *Drakaea gracilis* |  | |  | X |
| *Drakaea isolata* |  | |  | X |
| *Drakaea livida* |  | |  | X |
| *Drakaea micrantha* |  | |  | X |
| *Drakaea thynniphila* |  | |  | X |
| *Drymoanthus minutus* |  | |  | X |
| *Elythranthera brunonis* |  | |  | X |
| *Elythranthera emarginata* |  | |  | X |
| *Elythranthera intermedia* |  | |  | X |
| *Empusa habenarina* |  | |  | X |
| *Epiblema grandiflorum* |  | |  | X |
| *Epipogium roseum* |  | |  | X |
| *Eria eriaeoides* |  | |  | X |
| *Eria fitzalanii* |  | |  | X |
| *Eria inornata* |  | |  | X |
| *Eria intermedia* |  | |  | X |
| *Eria irukandjiana* | X | |  |  |
| *Eria kingii* |  | |  | X |
| *Eria queenslandica* |  | |  | X |
| *Ericksonella saccharata* |  | |  | X |
| *Eriochilus autumnalis* |  | |  | X |
| *Eriochilus cucullatus* |  | |  | X |
| *Eriochilus dilatatus* |  | |  | X |
| *Eriochilus helonomos* |  | |  | X |
| *Eriochilus magenteus* |  | |  | X |
| *Eriochilus multiflorus* |  | |  | X |
| *Eriochilus petricola* |  | |  | X |
| *Eriochilus pulchellus* |  | |  | X |
| *Eriochilus scaber* |  | |  | X |
| *Eriochilus* sp. *Swamp* (D.E.Murfet 1950b) D.L.Jones |  | |  | X |
| *Eriochilus tenuis* | X | |  |  |
| *Eriochilus valens* |  | |  | X |
| *Erythrorchis cassythoides* |  | |  | X |
| *Eucosia umbrosa* |  | |  | X |
| *Eulophia bicallosa* |  | | X |  |
| *Eulophia pelorica* |  | |  | X |
| *Eulophia venosa* |  | | X |  |
| *Eulophia zollingeri* |  | |  | X |
| *Flickingeria comata* |  | |  | X |
| *Flickingeria convexa* |  | | X |  |
| *Galeola cassythoides* |  | | X |  |
| *Galeola foliata* |  | |  | X |
| *Gastrodia crebriflora* |  | |  | X |
| *Gastrodia entomogama* |  | | X |  |
| *Gastrodia lacista* |  | | X |  |
| *Gastrodia procera* |  | |  | X |
| *Gastrodia queenslandica* |  | |  | X |
| *Gastrodia sesamoides* | X | |  |  |
| *Gastrodia surcula* |  | |  | X |
| *Gastrodia urceolata* |  | | X |  |
| *Gastrodia vescula* | X | |  |  |
| *Genoplesium acuminatum* |  | |  | X |
| *Genoplesium alticola* |  | |  | X |
| *Genoplesium alticolum* |  | |  | X |
| *Genoplesium apostasioides* |  | |  | X |
| *Genoplesium archeri* |  | |  | X |
| *Genoplesium arrectum* |  | |  | X |
| *Genoplesium baueri* |  | |  | X |
| *Genoplesium bishopii* |  | |  | X |
| *Genoplesium brachystachyum* |  | | X |  |
| *Genoplesium ciliatum* |  | |  | X |
| *Genoplesium citriodorum* |  | |  | X |
| *Genoplesium confertum* |  | |  | X |
| *Genoplesium cranei* |  | |  | X |
| *Genoplesium despectans* |  | |  | X |
| *Genoplesium eriochilum* |  | |  | X |
| *Genoplesium filiforme* |  | |  | X |
| *Genoplesium fimbriatum* |  | |  | X |
| *Genoplesium firthii* |  | |  | X |
| *Genoplesium formosum* |  | |  | X |
| *Genoplesium insignis* |  | | X |  |
| *Genoplesium littoralis* |  | |  | X |
| *Genoplesium morinum* | X | |  |  |
| *Genoplesium morrisii* |  | |  | X |
| *Genoplesium nigricans* |  | |  | X |
| *Genoplesium nudiscapum* |  | |  | X |
| *Genoplesium nudum* |  | |  | X |
| *Genoplesium oliganthum* |  | |  | X |
| *Genoplesium ostrinum* |  | |  | X |
| *Genoplesium parvicallum* | X | |  |  |
| *Genoplesium pedersonii* |  | |  | X |
| *Genoplesium plumosum* |  | |  | X |
| *Genoplesium psammophilum* | X | |  |  |
| *Genoplesium pumilum* |  | |  | X |
| *Genoplesium rhyoliticum* |  | |  | X |
| *Genoplesium rufum* | X | |  |  |
| *Genoplesium ruppii* |  | |  | X |
| *Genoplesium sagittiferum* |  | |  | X |
| *Genoplesium sigmoideum* |  | |  | X |
| *Genoplesium simulans* |  | |  | X |
| *Genoplesium* sp. aff. *systenum* (Brindabellas) |  | |  | X |
| *Genoplesium superbum* |  | | X |  |
| *Genoplesium systenum* |  | |  | X |
| *Genoplesium tasmanicum* |  | |  | X |
| *Genoplesium trifidum* |  | |  | X |
| *Genoplesium turfosum* |  | |  | X |
| *Genoplesium validum* |  | | X |  |
| *Genoplesium vernale* |  | | X |  |
| *Genoplesium woollsii* |  | |  | X |
| *Geodorum densiflorum* |  | |  | X |
| *Geodorum neocaledonicum* |  | |  | X |
| *Geodorum pictum* |  | |  | X |
| *Glossodia major* |  | |  | X |
| *Glossodia minor* |  | |  | X |
| *Goodyera grandis* |  | |  | X |
| *Goodyera rubicunda* |  | |  | X |
| *Goodyera viridiflora* | X | |  |  |
| *Grastidium baileyi* |  | |  | X |
| *Grastidium cancroides* |  | |  | X |
| *Grastidium luteocilium* |  | |  | X |
| *Grastidium tozerense* |  | |  | X |
| *Habenaria chlorosepala* |  | |  | X |
| *Habenaria divaricata* |  | | X |  |
| *Habenaria elongata* | X | |  |  |
| *Habenaria euryloba* |  | |  | X |
| *Habenaria eurystoma* | X | |  |  |
| *Habenaria exilis* | X | |  |  |
| *Habenaria ferdinandi* |  | | X |  |
| *Habenaria fuscina* |  | |  | X |
| *Habenaria halata* |  | |  | X |
| *Habenaria harroldii* |  | |  | X |
| *Habenaria holtzei* | X | |  |  |
| *Habenaria hymenophylla* |  | |  | X |
| *Habenaria macraithii* |  | |  | X |
| *Habenaria ochroleuca* |  | | X |  |
| *Habenaria papuana* |  | |  | X |
| *Habenaria praecox* |  | | X |  |
| *Habenaria propinquior* |  | |  | X |
| *Habenaria rumphii* |  | |  | X |
| *Habenaria* sp. *1* |  | |  | X |
| *Habenaria triplonema* |  | |  | X |
| *Habenaria xanthantha* |  | |  | X |
| *Leporella fimbriata* |  | |  | X |
| *Leptoceras fimbriata* |  | |  | X |
| *Leptoceras menziesii* | X | |  |  |
| *Liparis angustilabris* |  | |  | X |
| *Liparis bracteata* |  | |  | X |
| *Liparis coelogynoides* |  | |  | X |
| *Liparis collinsii* |  | |  | X |
| *Liparis cuneilabris* |  | | X |  |
| *Liparis fleckeri* | X | |  |  |
| *Liparis habenarina* |  | | X |  |
| *Liparis nugentiae* |  | |  | X |
| *Liparis reflexa* |  | |  | X |
| *Liparis simmondsii* |  | |  | X |
| *Liparis swenssonii* |  | |  | X |
| *Luisia atacta* |  | |  | X |
| *Luisia corrugata* |  | |  | X |
| *Luisia teretifolia* |  | |  | X |
| *Lyperanthus forrestii* |  | |  | X |
| *Lyperanthus nigricans* |  | |  | X |
| *Lyperanthus serratus* |  | |  | X |
| *Lyperanthus suaveolens* |  | |  | X |
| *Malaxis acuminata* |  | |  | X |
| *Malaxis fimbriata* |  | |  | X |
| *Malaxis latifolia* |  | |  | X |
| *Malaxis marsupichila* |  | |  | X |
| *Malaxis xanthochila* |  | |  | X |
| *Micropera fasciculata* |  | |  | X |
| *Microtis alba* |  | |  | X |
| *Microtis alboviridis* |  | |  | X |
| *Microtis angusii* |  | |  | X |
| *Microtis arenaria* |  | |  | X |
| *Microtis atrata* |  | |  | X |
| *Microtis benthamiana* |  | |  | X |
| *Microtis biloba* |  | |  | X |
| *Microtis bipulvinaris* |  | |  | X |
| *Microtis brownii* | X | |  |  |
| *Microtis cupularis* |  | |  | X |
| *Microtis densiflora* |  | |  | X |
| *Microtis eremaea* |  | |  | X |
| *Microtis eremicola* |  | |  | X |
| *Microtis familiaris* |  | |  | X |
| *Microtis frutetorum* |  | |  | X |
| *Microtis globula* |  | |  | X |
| *Microtis graniticola* |  | |  | X |
| *Microtis magnadenia* |  | |  | X |
| *Microtis media* | X | |  |  |
| *Microtis oblonga* |  | |  | X |
| *Microtis orbicularis* |  | |  | X |
| *Microtis parviflora* |  | |  | X |
| *Microtis pulchella* |  | |  | X |
| *Microtis quadrata* |  | |  | X |
| *Microtis rara* |  | |  | X |
| *Microtis truncata* |  | |  | X |
| *Microtis unifolia* |  | |  | X |
| *Mobilabium hamatum* |  | |  | X |
| *Monadenia micrantha* |  | |  | X |
| *Nervilia aragoana* | X | |  |  |
| *Nervilia crociformis* |  | |  | X |
| *Nervilia dallachyana* |  | |  | X |
| *Nervilia holochila* |  | |  | X |
| *Nervilia peltata* |  | |  | X |
| *Nervilia plicata* |  | |  | X |
| *Nervilia uniflora* |  | |  | X |
| *Oberonia attenuata* |  | |  | X |
| *Oberonia carnosa* |  | | X |  |
| *Oberonia complanata* |  | | X |  |
| *Oberonia flavescens* |  | |  | X |
| *Oberonia muelleriana* |  | | X |  |
| *Oberonia palmicola* |  | |  | X |
| *Oberonia rimachila* |  | |  | X |
| *Oberonia titania* |  | | X |  |
| *Octarrhena pusilla* |  | |  | X |
| *Orthoceras strictum* |  | |  | X |
| *Pachystoma pubescens* |  | |  | X |
| *Papillilabium beckleri* |  | |  | X |
| *Paracaleana alcockii* |  | |  | X |
| *Paracaleana brockmanii* | X | |  |  |
| *Paracaleana disjuncta* |  | |  | X |
| *Paracaleana dixonii* |  | |  | X |
| *Paracaleana gracilicordata* |  | |  | X |
| *Paracaleana granitica* |  | |  | X |
| *Paracaleana hortiorum* |  | |  | X |
| *Paracaleana lyonsii* |  | |  | X |
| *Paracaleana minor* |  | |  | X |
| *Paracaleana nigrita* |  | |  | X |
| *Paracaleana parvula* |  | |  | X |
| *Paracaleana sullivanii* |  | |  | X |
| *Paracaleana terminalis* |  | |  | X |
| *Paracaleana triens* |  | |  | X |
| *Parasarcochilus spathulatus* |  | |  | X |
| *Peristeranthus hillii* |  | |  | X |
| *Peristylus banfieldii* |  | |  | X |
| *Peristylus candidus* |  | |  | X |
| *Peristylus chlorandrellus* |  | | X |  |
| *Peristylus papuanus* |  | |  | X |
| *Phaius amboinensis* |  | |  | X |
| *Phaius australis* | X | |  |  |
| *Phaius bernaysii* |  | |  | X |
| *Phaius pictus* |  | |  | X |
| *Phaius tancarvilleae* |  | |  | X |
| *Phaius terrestre* |  | |  | X |
| *Phalaenopsis rosenstromii* | X | |  |  |
| *Pheladenia deformis* |  | |  | X |
| *Pholidota imbricata* |  | |  | X |
| *Pholidota pallida* |  | |  | X |
| *Plectorrhiza brevilabris* |  | |  | X |
| *Plectorrhiza tridentata* |  | |  | X |
| *Plexaure crassiuscula* |  | |  | X |
| *Pomatocalpa macphersonii* |  | |  | X |
| *Pomatocalpa marsupiale* |  | |  | X |
| *Praecoxanthus aphyllus* |  | |  | X |
| *Prasophyllum acuminatum* |  | |  | X |
| *Prasophyllum* aff. *montanum* B | X | |  |  |
| *Prasophyllum affine* |  | |  | X |
| *Prasophyllum albiglans* |  | |  | X |
| *Prasophyllum album* |  | | X |  |
| *Prasophyllum alpestre* |  | |  | X |
| *Prasophyllum alpinum* |  | |  | X |
| *Prasophyllum amoenum* |  | |  | X |
| *Prasophyllum anomalum* |  | | X |  |
| *Prasophyllum anticum* |  | |  | X |
| *Prasophyllum apoxychilum* |  | |  | X |
| *Prasophyllum archeri* |  | |  | X |
| *Prasophyllum atratum* |  | |  | X |
| *Prasophyllum australe* |  | | X |  |
| *Prasophyllum bagoensis* |  | |  | X |
| *Prasophyllum barnettii* | X | |  |  |
| *Prasophyllum beatrix* |  | |  | X |
| *Prasophyllum beaugleholei* |  | |  | X |
| *Prasophyllum bowdenae* | X | |  |  |
| *Prasophyllum brainei* |  | |  | X |
| *Prasophyllum brevilabre* |  | |  | X |
| *Prasophyllum brownii* |  | |  | X |
| *Prasophyllum calcicola* |  | |  | X |
| *Prasophyllum campestre* |  | |  | X |
| *Prasophyllum canaliculatum* |  | |  | X |
| *Prasophyllum candidum* |  | |  | X |
| *Prasophyllum caricetum* |  | |  | X |
| *Prasophyllum castaneum* |  | |  | X |
| *Prasophyllum catenemum* |  | |  | X |
| *Prasophyllum caudiculum* | X | |  |  |
| *Prasophyllum chasmogamum* | X | |  |  |
| *Prasophyllum colemaniae* |  | |  | X |
| *Prasophyllum collinum* | X | |  |  |
| *Prasophyllum concinnum* |  | |  | X |
| *Prasophyllum constrictum* |  | |  | X |
| *Prasophyllum correctum* |  | |  | X |
| *Prasophyllum crebriflorum* |  | |  | X |
| *Prasophyllum cucullatum* |  | |  | X |
| *Prasophyllum cyphochilum* |  | |  | X |
| *Prasophyllum densum* |  | |  | X |
| *Prasophyllum despectans* |  | |  | X |
| *Prasophyllum diversiflorum* |  | | X |  |
| *Prasophyllum dixonii* |  | |  | X |
| *Prasophyllum dossenum* | X | |  |  |
| *Prasophyllum drummondii* |  | |  | X |
| *Prasophyllum elatum* |  | |  | X |
| *Prasophyllum ellipticum* |  | |  | X |
| *Prasophyllum eriochilum* | X | |  |  |
| *Prasophyllum erythrocommum* |  | | X |  |
| *Prasophyllum exilis* | X | |  |  |
| *Prasophyllum favonium* |  | |  | X |
| *Prasophyllum fecundum* |  | |  | X |
| *Prasophyllum filiforme* |  | |  | X |
| *Prasophyllum fimbria* |  | |  | X |
| *Prasophyllum fimbriatum* |  | |  | X |
| *Prasophyllum fitzgeraldii* |  | |  | X |
| *Prasophyllum flavum* |  | |  | X |
| *Prasophyllum fosteri* |  | |  | X |
| *Prasophyllum frenchii* | X | |  |  |
| *Prasophyllum fuscum* |  | |  | X |
| *Prasophyllum gibbosum* |  | |  | X |
| *Prasophyllum giganteum* |  | |  | X |
| *Prasophyllum gilgai* |  | |  | X |
| *Prasophyllum goldsackii* | X | |  |  |
| *Prasophyllum gracile* |  | |  | X |
| *Prasophyllum gracillimum* |  | |  | X |
| *Prasophyllum grimwadeanum* | X | |  |  |
| *Prasophyllum helophilum* | X | |  |  |
| *Prasophyllum hians* |  | | X |  |
| *Prasophyllum hopsonii* |  | |  | X |
| *Prasophyllum hygrophilum* |  | | X |  |
| *Prasophyllum incompositum* |  | |  | X |
| *Prasophyllum incorrectum* |  | |  | X |
| *Prasophyllum incurvum* |  | |  | X |
| *Prasophyllum innubum* |  | |  | X |
| *Prasophyllum keltonii* |  | |  | X |
| *Prasophyllum laminatum* | X | |  |  |
| *Prasophyllum lanceolatum* | X | |  |  |
| *Prasophyllum laxum* | X | |  |  |
| *Prasophyllum limnetes* | X | |  |  |
| *Prasophyllum lindleyanum* |  | |  | X |
| *Prasophyllum litorale* |  | |  | X |
| *Prasophyllum longisepalum* |  | |  | X |
| *Prasophyllum maccannii* |  | | X |  |
| *Prasophyllum macrostachyum* |  | |  | X |
| *Prasophyllum macrotys* |  | |  | X |
| *Prasophyllum milfordense* |  | |  | X |
| *Prasophyllum mimulum* | X | |  |  |
| *Prasophyllum mollissimum* |  | |  | X |
| *Prasophyllum montanum* |  | | X |  |
| *Prasophyllum morganii* |  | |  | X |
| *Prasophyllum morrisii* |  | |  | X |
| *Prasophyllum mucronatum* |  | |  | X |
| *Prasophyllum muelleri* | X | |  |  |
| *Prasophyllum murfetii* | X | |  |  |
| *Prasophyllum nigricans* |  | |  | X |
| *Prasophyllum niphopedium* |  | |  | X |
| *Prasophyllum nublingii* |  | |  | X |
| *Prasophyllum nudiscapum* |  | | X |  |
| *Prasophyllum nudum* | X | |  |  |
| *Prasophyllum occidentale* | X | |  |  |
| *Prasophyllum occultans* |  | |  | X |
| *Prasophyllum odoratissimum* |  | |  | X |
| *Prasophyllum odoratum* |  | |  | X |
| *Prasophyllum olidum* |  | |  | X |
| *Prasophyllum ovale* |  | |  | X |
| *Prasophyllum pallens* |  | |  | X |
| *Prasophyllum pallidum* |  | | X |  |
| *Prasophyllum parviflorum* |  | |  | X |
| *Prasophyllum parvifolium* |  | |  | X |
| *Prasophyllum patens* |  | |  | X |
| *Prasophyllum paulinae* |  | |  | X |
| *Prasophyllum perangustum* |  | |  | X |
| *Prasophyllum petilum* | X | |  |  |
| *Prasophyllum plumaeforme* |  | |  | X |
| *Prasophyllum plumiforme* |  | |  | X |
| *Prasophyllum praecox* |  | |  | X |
| *Prasophyllum pruinosum* | X | |  |  |
| *Prasophyllum pulchellum* |  | |  | X |
| *Prasophyllum pumilum* |  | |  | X |
| *Prasophyllum pyriforme* |  | |  | X |
| *Prasophyllum readii* |  | |  | X |
| *Prasophyllum regium* | X | |  |  |
| *Prasophyllum retroflexum* |  | |  | X |
| *Prasophyllum robustum* |  | |  | X |
| *Prasophyllum rogersii* | X | |  |  |
| *Prasophyllum rostratum* |  | |  | X |
| *Prasophyllum rotundiflorum* |  | |  | X |
| *Prasophyllum rufum* | X | |  |  |
| *Prasophyllum ruppii* | X | |  |  |
| *Prasophyllum sargentii* |  | |  | X |
| *Prasophyllum secutum* |  | |  | X |
| *Prasophyllum solstitium* |  | |  | X |
| *Prasophyllum* sp. aff. *brevilabre* (South-western Victoria) | X | |  |  |
| *Prasophyllum* sp. aff. *fitzgeraldii* D | X | |  |  |
| *Prasophyllum* sp. aff. *frenchii* 3 | X | |  |  |
| *Prasophyllum* sp. aff. *occidentale* E | X | |  |  |
| *Prasophyllum* sp. aff. *odoratum* A | X | |  |  |
| *Prasophyllum* sp. aff. *suttonii* (Boyd Plateau) | X | |  |  |
| *Prasophyllum* sp. Coast sandhills (Hj.Eichler 14100) R.J.Bates |  | | X |  |
| *Prasophyllum* sp. Enigma (R.Bates 2350) R.J.Bates |  | |  | X |
| *Prasophyllum sphacelatum* | X | |  |  |
| *Prasophyllum spicatum* |  | |  | X |
| *Prasophyllum stellatum* |  | |  | X |
| *Prasophyllum striatum* |  | |  | X |
| *Prasophyllum suaveolens* |  | |  | X |
| *Prasophyllum subbisectum* |  | |  | X |
| *Prasophyllum suttonii* |  | |  | X |
| *Prasophyllum sylvestre* |  | |  | X |
| *Prasophyllum tadgellianum* |  | |  | X |
| *Prasophyllum taphanyx* |  | |  | X |
| *Prasophyllum triangulare* | X | |  |  |
| *Prasophyllum trifidum* |  | |  | X |
| *Prasophyllum tunbridgense* |  | |  | X |
| *Prasophyllum unicum* |  | |  | X |
| *Prasophyllum uvidulum* | X | |  |  |
| *Prasophyllum validum* |  | |  | X |
| *Prasophyllum venustum* |  | |  | X |
| *Prasophyllum viretrum* |  | | X |  |
| *Prasophyllum viride* |  | |  | X |
| *Prasophyllum wallum* |  | |  | X |
| *Prasophyllum wilkinsoniorum* | X | |  |  |
| *Prasophyllum wilsoniense* |  | |  | X |
| *Prasophyllum woollsii* |  | | X |  |
| *Pseudovanilla foliata* |  | | X |  |
| *Pteroceras spathulatus* |  | |  | X |
| *Pterostylis abrupta* |  | |  | X |
| *Pterostylis aciculiformis* |  | |  | X |
| *Pterostylis acuminata* |  | |  | X |
| *Pterostylis aestiva* |  | |  | X |
| *Pterostylis* aff. *barbata* |  | |  | X |
| *Pterostylis* aff. *nana* | X | |  |  |
| *Pterostylis* aff. *plumosa* |  | |  | X |
| *Pterostylis* aff. *rufa* |  | |  | X |
| *Pterostylis alata* |  | | X |  |
| *Pterostylis allantoidea* |  | |  | X |
| *Pterostylis alpina* |  | |  | X |
| *Pterostylis alveata* |  | |  | X |
| *Pterostylis anatona* |  | |  | X |
| *Pterostylis aneba* |  | |  | X |
| *Pterostylis angusta* |  | |  | X |
| *Pterostylis aphylla* |  | |  | X |
| *Pterostylis aquilonia* |  | |  | X |
| *Pterostylis arenicola* | X | |  |  |
| *Pterostylis aspera* |  | |  | X |
| *Pterostylis atrans* |  | |  | X |
| *Pterostylis atriola* |  | |  | X |
| *Pterostylis baptistii* |  | |  | X |
| *Pterostylis barbata* |  | |  | X |
| *Pterostylis barringtonensis* |  | |  | X |
| *Pterostylis basaltica* |  | |  | X |
| *Pterostylis bicolor* | X | |  |  |
| *Pterostylis bicornis* |  | |  | X |
| *Pterostylis biseta* |  | |  | X |
| *Pterostylis brevichila* |  | |  | X |
| *Pterostylis brevisepala* | X | |  |  |
| *Pterostylis bryophila* |  | |  | X |
| *Pterostylis calceolus* | X | |  |  |
| *Pterostylis celans* |  | |  | X |
| *Pterostylis chaetophora* | X | |  |  |
| *Pterostylis cheraphila* |  | |  | X |
| *Pterostylis chlorogramma* |  | |  | X |
| *Pterostylis chocolatina* |  | |  | X |
| *Pterostylis ciliata* |  | |  | X |
| *Pterostylis clavigera* |  | |  | X |
| *Pterostylis cobarensis* |  | |  | X |
| *Pterostylis coccina* |  | |  | X |
| *Pterostylis collina* |  | |  | X |
| *Pterostylis commutata* |  | |  | X |
| *Pterostylis concava* |  | |  | X |
| *Pterostylis concinna* |  | |  | X |
| *Pterostylis conferta* |  | |  | X |
| *Pterostylis conoglossa* | X | |  |  |
| *Pterostylis crassa* | X | |  |  |
| *Pterostylis crassicaulis* | X | |  |  |
| *Pterostylis crassichila* |  | | X |  |
| *Pterostylis crypta* |  | |  | X |
| *Pterostylis cucullata* | X | |  |  |
| *Pterostylis curta* |  | |  | X |
| *Pterostylis daintreana* |  | |  | X |
| *Pterostylis daintreeana* |  | |  | X |
| *Pterostylis decurva* |  | |  | X |
| *Pterostylis depauperata* |  | |  | X |
| *Pterostylis despectans* |  | |  | X |
| *Pterostylis dilatata* |  | |  | X |
| *Pterostylis diminuta* |  | |  | X |
| *Pterostylis dolichochila* | X | |  |  |
| *Pterostylis dubia* |  | |  | X |
| *Pterostylis elegans* |  | |  | X |
| *Pterostylis erecta* |  | |  | X |
| *Pterostylis erythroconcha* |  | |  | X |
| *Pterostylis exalla* |  | |  | X |
| *Pterostylis falcata* |  | |  | X |
| *Pterostylis ferruginea* |  | |  | X |
| *Pterostylis fischii* |  | |  | X |
| *Pterostylis flavovirens* |  | |  | X |
| *Pterostylis foliata* |  | |  | X |
| *Pterostylis frenchii* |  | |  | X |
| *Pterostylis furcata* |  | |  | X |
| *Pterostylis furcillata* |  | |  | X |
| *Pterostylis gibbosa* |  | |  | X |
| *Pterostylis gracilis* |  | |  | X |
| *Pterostylis grandiflora* | X | |  |  |
| *Pterostylis hamata* |  | |  | X |
| *Pterostylis hamiltonii* |  | |  | X |
| *Pterostylis hians* |  | |  | X |
| *Pterostylis hildae* | X | |  |  |
| *Pterostylis hispidula* |  | |  | X |
| *Pterostylis insectifera* |  | |  | X |
| *Pterostylis laxa* |  | |  | X |
| *Pterostylis lepida* |  | |  | X |
| *Pterostylis leptochila* | X | |  |  |
| *Pterostylis lineata* |  | |  | X |
| *Pterostylis lingua* |  | |  | X |
| *Pterostylis littoralis* |  | |  | X |
| *Pterostylis loganii* | X | |  |  |
| *Pterostylis longicurva* | X | |  |  |
| *Pterostylis longifolia* |  | |  | X |
| *Pterostylis longipetala* |  | |  | X |
| *Pterostylis lustra* |  | |  | X |
| *Pterostylis macilenta* |  | |  | X |
| *Pterostylis macrocalymma* |  | | X |  |
| *Pterostylis macrosepala* | X | |  |  |
| *Pterostylis major* |  | |  | X |
| *Pterostylis maxima* | X | |  |  |
| *Pterostylis melagramma* |  | |  | X |
| *Pterostylis metcalfei* |  | |  | X |
| *Pterostylis microglossa* |  | |  | X |
| *Pterostylis mirabilis* |  | |  | X |
| *Pterostylis mitchellii* | X | |  |  |
| *Pterostylis monticola* |  | |  | X |
| *Pterostylis multiflora* |  | |  | X |
| *Pterostylis mutica* |  | | X |  |
| *Pterostylis mystacina* |  | |  | X |
| *Pterostylis nana* |  | | X |  |
| *Pterostylis nigricans* |  | |  | X |
| *Pterostylis nutans* |  | |  | X |
| *Pterostylis oblonga* |  | |  | X |
| *Pterostylis ophioglossa* |  | |  | X |
| *Pterostylis oreophila* |  | |  | X |
| *Pterostylis ovata* |  | |  | X |
| *Pterostylis papuana* |  | |  | X |
| *Pterostylis parca* |  | | X |  |
| *Pterostylis parviflora* |  | |  | X |
| *Pterostylis patens* |  | |  | X |
| *Pterostylis pedoglossa* | X | |  |  |
| *Pterostylis pedunculata* |  | |  | X |
| *Pterostylis petrosa* |  | |  | X |
| *Pterostylis picta* |  | |  | X |
| *Pterostylis planulata* |  | |  | X |
| *Pterostylis plumosa* |  | |  | X |
| *Pterostylis praetermissa* |  | |  | X |
| *Pterostylis prasina* |  | |  | X |
| *Pterostylis pratensis* |  | |  | X |
| *Pterostylis procera* |  | |  | X |
| *Pterostylis psammophila* |  | |  | X |
| *Pterostylis pulchella* |  | |  | X |
| *Pterostylis pusilla* |  | |  | X |
| *Pterostylis pyramidalis* |  | |  | X |
| *Pterostylis recurva* |  | |  | X |
| *Pterostylis reflexa* |  | |  | X |
| *Pterostylis revoluta* |  | |  | X |
| *Pterostylis riparia* |  | |  | X |
| *Pterostylis robusta* |  | |  | X |
| *Pterostylis roensis* |  | |  | X |
| *Pterostylis rogersii* |  | |  | X |
| *Pterostylis rubenachii* |  | |  | X |
| *Pterostylis rubescens* |  | |  | X |
| *Pterostylis rufa* |  | |  | X |
| *Pterostylis russellii* |  | |  | X |
| *Pterostylis sanguinea* |  | |  | X |
| *Pterostylis sargentii* |  | |  | X |
| *Pterostylis saxicola* |  | |  | X |
| *Pterostylis scabra* |  | |  | X |
| *Pterostylis scabrida* |  | |  | X |
| *Pterostylis scoliosa* |  | |  | X |
| *Pterostylis setifera* |  | |  | X |
| *Pterostylis sinuata* |  | |  | X |
| *Pterostylis smaragdyna* | X | |  |  |
| *Pterostylis* sp. aff. *aciculiformis* (Stawell) |  | |  | X |
| *Pterostylis* sp. aff. *alata* (New England) | X | |  |  |
| *Pterostylis* sp. aff. *alveata* (Montane) | X | |  |  |
| *Pterostylis* sp. aff. *dolichochila* (Portland) | X | |  |  |
| *Pterostylis* sp. aff. *gibbosa* (Sydney) |  | | X |  |
| *Pterostylis* sp. aff. *hamata* (Northern Tablelands) | X | |  |  |
| *Pterostylis* sp. aff. *mutica* (Basalt Plains) | X | |  |  |
| *Pterostylis* sp. aff. *parviflora* (Ebor) | X | |  |  |
| *Pterostylis* sp. aff. *parviflora* (Southern Victoria) |  | | X |  |
| *Pterostylis* sp. aff. *plumosa* (Anglesea) | X | |  |  |
| *Pterostylis* sp. aff. *reflexa* (Tablelands) |  | |  | X |
| *Pterostylis* sp. aff. *russellii* (New England) | X | |  |  |
| *Pterostylis* sp. aff. *tunstallii* (Blue Mountains) | X | |  |  |
| *Pterostylis* sp. B | X | |  |  |
| *Pterostylis* sp. Coastal (R.Bates 37621) |  | |  | X |
| *Pterostylis* sp. Denman (ORG 5019) |  | |  | X |
| *Pterostylis* sp. Flat Rock Creek (D.L.Jones 15873 & K.J.Fitzgerald) |  | |  | X |
| *Pterostylis* sp. Gundiah (W.W.Abell AQ72188) |  | |  | X |
| *Pterostylis* sp. Hale (R.Bates 21725) | X | |  |  |
| *Pterostylis* sp. Joyners Ridge (R.Crane 17) |  | |  | X |
| *Pterostylis* sp. Mt Maroon (J.R.Clarkson+ 1114) |  | | X |  |
| *Pterostylis* sp. Rock ledges (pl. 185, Bates & Weber 1990) |  | | X |  |
| *Pterostylis* sp. Sandheath (D.Murfet 3190) |  | |  | X |
| *Pterostylis* sp. Triloba (pl. 191, Bates & Weber 1990) | X | |  |  |
| *Pterostylis spathulata* | X | |  |  |
| *Pterostylis stenochila* |  | |  | X |
| *Pterostylis stenosepala* |  | |  | X |
| *Pterostylis striata* |  | |  | X |
| *Pterostylis stricta* |  | |  | X |
| *Pterostylis subtilis* |  | |  | X |
| *Pterostylis tasmanica* |  | |  | X |
| *Pterostylis taurus* |  | |  | X |
| *Pterostylis tenuis* |  | |  | X |
| *Pterostylis tenuissima* | X | |  |  |
| *Pterostylis timothyi* |  | |  | X |
| *Pterostylis torquata* |  | |  | X |
| *Pterostylis toveyana* |  | |  | X |
| *Pterostylis truncata* | X | |  |  |
| *Pterostylis tunstallii* |  | |  | X |
| *Pterostylis turfosa* |  | |  | X |
| *Pterostylis uliginosa* |  | |  | X |
| *Pterostylis umbrina* |  | |  | X |
| *Pterostylis valida* |  | |  | X |
| *Pterostylis ventricosa* |  | |  | X |
| *Pterostylis vereenae* | X | |  |  |
| *Pterostylis vernalis* | X | |  |  |
| *Pterostylis viriosa* |  | |  | X |
| *Pterostylis vitrea* |  | |  | X |
| *Pterostylis vittata* | X | |  |  |
| *Pterostylis wapstrarum* |  | |  | X |
| *Pterostylis whitei* | X | |  |  |
| *Pterostylis williamsonii* | X | |  |  |
| *Pterostylis woollsii* |  | |  | X |
| *Pterostylis xerophila* |  | |  | X |
| *Pterostylis ziegeleri* |  | |  | X |
| *Pyrorchis forrestii* |  | |  | X |
| *Pyrorchis nigricans* |  | |  | X |
| *Rhinerrhiza divitiflora* |  | |  | X |
| *Rhinerrhizopsis matutina* |  | |  | X |
| *Rhipidorchis micrantha* |  | |  | X |
| *Rhizanthella gardneri* |  | |  | X |
| *Rhizanthella omissa* | X | |  |  |
| *Rhizanthella slateri* |  | | X |  |
| *Rimacola elliptica* |  | |  | X |
| *Robiquetia gracilistipes* |  | |  | X |
| *Robiquetia rectifolia* |  | |  | X |
| *Robiquetia wassellii* | X | |  |  |
| *Saccolabiopsis armitii* |  | |  | X |
| *Saccolabiopsis rectifolia* |  | |  | X |
| *Sarcanthopsis warocqueana* |  | |  | X |
| *Sarcochilus aequalis* | X | |  |  |
| *Sarcochilus argochilus* |  | |  | X |
| *Sarcochilus australis* |  | | X |  |
| *Sarcochilus borealis* |  | |  | X |
| *Sarcochilus ceciliae* |  | |  | X |
| *Sarcochilus dilatatus* |  | |  | X |
| *Sarcochilus eriochilus* |  | |  | X |
| *Sarcochilus falcatus* |  | |  | X |
| *Sarcochilus fitzgeraldii* |  | |  | X |
| *Sarcochilus hartmannii* |  | |  | X |
| *Sarcochilus hillii* |  | |  | X |
| *Sarcochilus hirticalcar* |  | |  | X |
| *Sarcochilus minutiflos* |  | |  | X |
| *Sarcochilus olivaceus* |  | |  | X |
| *Sarcochilus parviflorus* |  | |  | X |
| *Sarcochilus roseus* |  | |  | X |
| *Sarcochilus serrulatus* | X | |  |  |
| *Sarcochilus spathulatus* |  | |  | X |
| *Sarcochilus tricalliatus* |  | |  | X |
| *Sarcochilus tridentatus* |  | | X |  |
| *Sarcochilus weinthalii* |  | | X |  |
| *Schistotylus purpuratus* |  | |  | X |
| *Schoenorchis micrantha* |  | |  | X |
| *Schoenorchis sarcophylla* |  | |  | X |
| *Spathoglottis paulinae* |  | | X |  |
| *Spathoglottis plicata* |  | |  | X |
| *Spiculaea ciliata* |  | |  | X |
| *Spiculaea huntiana* |  | |  | X |
| *Spiculaea irritabilis* |  | | X |  |
| *Spiranthes alticola* |  | |  | X |
| *Spiranthes australis* |  | |  | X |
| *Spiranthes lancea* |  | |  | X |
| *Spiranthes sinensis* |  | |  | X |
| *Taeniophyllum confertum* |  | |  | X |
| *Taeniophyllum cymbiforme* |  | | X |  |
| *Taeniophyllum flavum* |  | |  | X |
| *Taeniophyllum glandulosum* |  | |  | X |
| *Taeniophyllum lobatum* |  | | X |  |
| *Taeniophyllum malianum* |  | |  | X |
| *Taeniophyllum muelleri* |  | |  | X |
| *Tainia parviflora* |  | |  | X |
| *Tainia trinervis* |  | |  | X |
| *Thelasis carinata* |  | |  | X |
| *Thelymitra adorata* |  | |  | X |
| *Thelymitra* aff. *longifolia* | X | |  |  |
| *Thelymitra* aff. *pauciflora* |  | | X |  |
| *Thelymitra aggericola* |  | |  | X |
| *Thelymitra albiflora* |  | |  | X |
| *Thelymitra alpicola* |  | |  | X |
| *Thelymitra angustifolia* |  | |  | X |
| *Thelymitra antennifera* |  | |  | X |
| *Thelymitra apiculata* |  | |  | X |
| *Thelymitra arenaria* | X | |  |  |
| *Thelymitra aristata* |  | |  | X |
| *Thelymitra atronitida* |  | |  | X |
| *Thelymitra basaltica* |  | |  | X |
| *Thelymitra batesii* | X | |  |  |
| *Thelymitra benthamiana* |  | |  | X |
| *Thelymitra bracteata* |  | |  | X |
| *Thelymitra brevifolia* |  | |  | X |
| *Thelymitra campanulata* |  | |  | X |
| *Thelymitra canaliculata* |  | |  | X |
| *Thelymitra carnea* |  | |  | X |
| *Thelymitra chasmogama* |  | |  | X |
| *Thelymitra circumsepta* |  | |  | X |
| *Thelymitra cornicina* |  | |  | X |
| *Thelymitra crinita* |  | |  | X |
| *Thelymitra cucullata* |  | |  | X |
| *Thelymitra cyanapicata* |  | |  | X |
| *Thelymitra cyanea* | X | |  |  |
| *Thelymitra decora* |  | |  | X |
| *Thelymitra dedmaniarum* |  | | X |  |
| *Thelymitra epipactoides* |  | |  | X |
| *Thelymitra erosa* |  | |  | X |
| *Thelymitra exigua* |  | |  | X |
| *Thelymitra fasciculata* |  | |  | X |
| *Thelymitra flexuosa* |  | | X |  |
| *Thelymitra fragrans* |  | |  | X |
| *Thelymitra frenchii* |  | |  | X |
| *Thelymitra fuscolutea* | X | |  |  |
| *Thelymitra graminea* |  | |  | X |
| *Thelymitra grandiflora* |  | |  | X |
| *Thelymitra granitora* |  | |  | X |
| *Thelymitra gregaria* |  | |  | X |
| *Thelymitra hiemalis* | X | |  |  |
| *Thelymitra holmesii* |  | |  | X |
| *Thelymitra hygrophila* |  | |  | X |
| *Thelymitra imbricata* | X | |  |  |
| *Thelymitra improcera* |  | |  | X |
| *Thelymitra incurva* |  | |  | X |
| *Thelymitra inflata* |  | |  | X |
| *Thelymitra irregularis* |  | |  | X |
| *Thelymitra ixioides* |  | |  | X |
| *Thelymitra jacksonii* |  | |  | X |
| *Thelymitra jonesii* |  | |  | X |
| *Thelymitra juncifolia* |  | |  | X |
| *Thelymitra kangaloonica* |  | |  | X |
| *Thelymitra latifolia* |  | |  | X |
| *Thelymitra latiloba* |  | |  | X |
| *Thelymitra longifolia* |  | |  | X |
| *Thelymitra longiloba* |  | |  | X |
| *Thelymitra lucida* |  | |  | X |
| *Thelymitra luteocilium* |  | |  | X |
| *Thelymitra mackibbinii* |  | |  | X |
| *Thelymitra macmillanii* |  | |  | X |
| *Thelymitra macrophylla* |  | |  | X |
| *Thelymitra maculata* |  | |  | X |
| *Thelymitra magnifica* |  | |  | X |
| *Thelymitra malvina* | X | |  |  |
| *Thelymitra matthewsii* |  | |  | X |
| *Thelymitra media* |  | |  | X |
| *Thelymitra megcalyptra* |  | |  | X |
| *Thelymitra merraniae* |  | |  | X |
| *Thelymitra mucida* |  | |  | X |
| *Thelymitra nuda* |  | |  | X |
| *Thelymitra occidentalis* |  | |  | X |
| *Thelymitra odora* |  | |  | X |
| *Thelymitra orientalis* |  | |  | X |
| *Thelymitra pallidiflora* |  | | X |  |
| *Thelymitra pallidifructus* |  | |  | X |
| *Thelymitra paludosa* |  | |  | X |
| *Thelymitra peniculata* |  | |  | X |
| *Thelymitra petrophila* |  | |  | X |
| *Thelymitra planicola* |  | |  | X |
| *Thelymitra polychroma* |  | |  | X |
| *Thelymitra psammophila* |  | |  | X |
| *Thelymitra pulchella* | X | |  |  |
| *Thelymitra pulcherrima* |  | |  | X |
| *Thelymitra purpurata* | X | |  |  |
| *Thelymitra reflexa* |  | |  | X |
| *Thelymitra retecta* | X | |  |  |
| *Thelymitra rubra* |  | |  | X |
| *Thelymitra rubricaulis* |  | |  | X |
| *Thelymitra sargentii* |  | |  | X |
| *Thelymitra silena* |  | |  | X |
| *Thelymitra simulata* |  | |  | X |
| *Thelymitra* sp. Black buds (R.Bates 64389) |  | |  | X |
| *Thelymitra spadicea* | X | |  |  |
| *Thelymitra sparsa* | X | |  |  |
| *Thelymitra speciosa* |  | |  | X |
| *Thelymitra spiralis* |  | |  | X |
| *Thelymitra stellata* |  | |  | X |
| *Thelymitra tigrina* |  | |  | X |
| *Thelymitra truncata* |  | |  | X |
| *Thelymitra uliginosa* |  | |  | X |
| *Thelymitra variegata* |  | |  | X |
| *Thelymitra venosa* |  | |  | X |
| *Thelymitra villosa* |  | |  | X |
| *Thelymitra viridis* |  | |  | X |
| *Thelymitra vulgaris* |  | |  | X |
| *Thelymitra xanthotricha* |  | |  | X |
| *Thelymitra yorkensis* |  | |  | X |
| *Thrixspermum album* | X | |  |  |
| *Thrixspermum congestum* |  | | X |  |
| *Thrixspermum platystachys* | X | |  |  |
| *Thynninorchis huntiana* |  | |  | X |
| *Thynninorchis huntianus* |  | |  | X |
| *Townsonia viridis* |  | |  | X |
| *Trachoma papuanum* |  | | X |  |
| *Trachoma speciosum* |  | |  | X |
| *Trachoma stellatum* |  | |  | X |
| *Trichoglottis australiensis* |  | |  | X |
| *Tropidia curculigoides* |  | |  | X |
| *Tropidia territorialis* |  | |  | X |
| *Vanda hindsii* |  | |  | X |
| *Vrydagzynea grayi* |  | |  | X |
| *Zeuxine oblonga* |  | |  | X |
| *Zeuxine polygonoides* |  | |  | X |

**Appendix S2.1:** Australia’s ten most diverse angiosperm families according to cleaned herbarium records extracted from Australia’s Virtual Herbarium.

| Family | n species in family | n records for family | Mean records per species |
| --- | --- | --- | --- |
| Fabaceae | 2767 | 890762 | 322 |
| Myrtaceae | 2396 | 926457 | 387 |
| Orchidaceae | 1540 | 174601 | 113 |
| Proteaceae | 1168 | 311467 | 267 |
| Asteraceae | 1097 | 488471 | 445 |
| Poaceae | 1080 | 738536 | 684 |
| Cyperaceae | 802 | 283250 | 353 |
| Malvaceae | 652 | 110988 | 170 |
| Ericaceae | 585 | 196232 | 335 |
| Rutaceae | 497 | 119957 | 241 |

**Appendix S2.2:** Australia’s most diverse orchid genera according to cleaned herbarium records extracted from Australia’s Virtual Herbarium.

| Genus | Species in genus | n records per genus | Mean records per species |
| --- | --- | --- | --- |
| *Caladenia* | 279 | 29144 | 104.5 |
| *Pterostylis* | 208 | 29494 | 141.8 |
| *Prasophyllum* | 164 | 12886 | 78.6 |
| *Thelymitra* | 110 | 16624 | 151.1 |
| *Diuris* | 96 | 11933 | 124.3 |
| *Dendrobium* | 61 | 6222 | 102.0 |
| *Genoplesium* | 48 | 1854 | 38.6 |
| *Corunastylis* | 44 | 2468 | 56.1 |
| *Bulbophyllum* | 39 | 1481 | 38.0 |
| *Chiloglottis* | 31 | 4276 | 137.9 |
| *Calochilus* | 30 | 3265 | 108.8 |
| *Microtis* | 27 | 7120 | 263.7 |
| *Corybas* | 23 | 2702 | 117.5 |
| *Habenaria* | 21 | 522 | 24.9 |
| *Sarcochilus* | 21 | 2066 | 98.4 |
| *Dockrillia* | 18 | 748 | 41.6 |
| *Acianthus* | 15 | 4584 | 305.6 |
| *Arthrochilus* | 14 | 512 | 36.6 |
| *Paracaleana* | 14 | 668 | 47.7 |
| *Dipodium* | 12 | 3232 | 269.3 |
| *Eriochilus* | 12 | 3116 | 259.7 |
| *Cyanicula* | 11 | 1459 | 132.6 |
| *Liparis* | 11 | 398 | 36.2 |
| *Drakaea* | 10 | 728 | 72.8 |

*The remaining genera (n = 96) each have less than 10 species: *Acriopsis, Adenochilus, Anoectochilus, Aphyllorchis, Apostasia, Appendicula, Bromheadia, Burnettia, Cadetia, Calanthe, Caleana, Cheirostylis, Chiloschista, Cooktownia, Corymborkis, Corysanthes, Crepidium, Cryptostylis, Cymbidium, Cyrtostylis, Danhatchia, Demorchis, Didymoplexis, Dienia, Diplocaulobium, Diteilis, Drymoanthus, Elythranthera, Empusa, Epiblema, Epipogium, Eria, Ericksonella, Erythrorchis, Eucosia, Eulophia, Flickingeria, Galeola, Gastrodia, Geodorum, Glossodia, Goodyera, Grastidium, Leporella, Leptoceras, Luisia, Lyperanthus, Malaxis, Micropera, Mobilabium, Monadenia, Nervilia, Oberonia, Octarrhena, Orthoceras, Pachystoma, Papillilabium, Parasarcochilus, Peristeranthus, Peristylus, Phaius, Phalaenopsis, Pheladenia, Pholidota, Plectorrhiza, Plexaure, Pomatocalpa, Praecoxanthus, Pseudovanilla, Pteroceras, Pyrorchis, Rhinerrhiza, Rhinerrhizopsis, Rhipidorchis, Rhizanthella, Rimacola, Robiquetia, Saccolabiopsis, Sarcanthopsis, Schistotylus, Schoenorchis, Spathoglottis, Spiculaea, Spiranthes, Taeniophyllum, Tainia, Thelasis, Thrixspermum, Thynninorchis, Townsonia, Trachoma, Trichoglottis, Tropidia, Vanda, Vrydagzynea, Zeuxine*
